# Enhancing Cancer Therapy with Hyperthermia: Synergistic Effects with Natural Compounds and Conventional Treatments

**DOI:** 10.3390/ijms27041650

**Published:** 2026-02-08

**Authors:** Nada Oršolić, Darko Kučan, Maja Jazvinšćak Jembrek

**Affiliations:** 1 Division of Animal Physiology, Faculty of Science, University of Zagreb, Rooseveltov Trg 6, 10000 Zagreb, Croatia; 2 Division of Abdominal Surgery and Organ Transplantation, Department of Surgery, University Hospital Merkur, Zajčeva 19, 10000 Zagreb, Croatia; dkucan@gmail.com; 3 Division of Molecular Medicine, Laboratory for Protein Dynamics, Ruđer Bošković Institute, Bijenička Cesta 54, 10000 Zagreb, Croatia; maja.jazvinscak.jembrek@irb.hr; 4 School of Medicine, Catholic University of Croatia, Ilica 244, 10000 Zagreb, Croatia

**Keywords:** hyperthermia, combination cancer treatment with chemotherapy and/or radiotherapy, natural compounds, flavonoids, heat shock proteins, apoptosis, DNA damage, ROS, tumor microenvironment

## Abstract

Hyperthermia (HT) is a promising adjunct to conventional cancer therapies such as chemotherapy and radiotherapy (RT). It offers several advantages, including low toxicity to normal tissues, limited tumor resistance, and synergistic therapeutic effects. HT enhances treatment efficacy by inhibiting DNA repair mechanisms, increasing tumor membrane permeability for improved drug uptake, and improving oxygenation to reduce hypoxia-induced resistance. HT also promotes cancer cell death by inducing oxidative stress, leading to mitochondrial dysfunction, caspase activation, and PARP cleavage. It causes G2/M cell cycle arrest and damages tumor vasculature. Additionally, HT downregulates proangiogenic and invasive factors such as TGF-β1, VEGF, and MMP-2/9, contributing to reduced tumor progression. Combining HT with natural compounds like propolis and flavonoids further improves therapeutic outcomes. These natural agents are accessible, cost-effective, and exhibit multi-targeted anticancer activity. In synergy with HT, they enhance reactive oxygen species (ROS) generation, suppress heat shock proteins, modulate the tumor microenvironment (TME), and activate immune responses. They may also reduce the side effects of conventional therapies and support tissue regeneration. Overall, HT, especially in combination with natural compounds, offers a multifaceted and effective approach to cancer therapy.

## 1. Hyperthermia

Hyperthermia (HT) refers to a condition of elevated body temperature. It may result from pathological processes such as bacteremia or viremia, or from external thermal stress. In clinical settings, HT is applied intentionally as a therapeutic procedure in which the whole body or a specific region is exposed to elevated temperatures, typically between 39 °C and 45 °C. Within this temperature range, tumor cells are more susceptible to heat-induced injury, whereas normal, healthy tissues remain largely unaffected.

Over the past two decades, HT has been successfully used as an adjunctive therapy in combination with standard chemotherapy and radiotherapy (RT). At higher temperatures, usually above 60 °C, HT is used in thermoablation procedures to directly destroy tumor tissue [1].

HT is considered a promising anticancer strategy and is most often applied alongside RT, chemotherapy, gene therapy, surgery, and immunotherapy. The key advantages of HT include its synergistic interaction with chemotherapy and RT, low toxicity toward normal tissues, and minimal development of resistance in tumor cells. Beyond its direct cytotoxicity, HT also exerts indirect antitumor effects by stimulating the immune system.

The therapeutic efficacy of HT depends on both temperature and duration of exposure [2,3]. Biologically effectiveness is generally observed between 40 °C and 45 °C. At temperatures above 43 °C, the required exposure time decreases by approximately half with each additional degree, while maintaining similar cytotoxic efficacy. When temperatures do not exceed 44 °C, normal tissues typically remain unaffected. However, exposure to temperatures above 45 °C may lead to irreversible damage to healthy tissues [1,2,3,4].

The primary mechanism of heat-induced cell death involves protein denaturation, which begins at temperatures above 40 °C. Additional mechanisms relevant to combined HT therapies include increased production of reactive oxygen species (ROS), DNA damage, cell cycle arrest, and mitochondrial membrane potential (MMP) depolarization, processes closely linked to elevated ROS levels. Excess ROS generated during heat stress can trigger nonspecific modifications of proteins, lipids, and nucleic acids, ultimately resulting in bioenergetic failure. Heat-affected proteins may be involved in cell division, energy metabolism, cytoskeletal organization, protein folding (chaperones), transcription, and protein synthesis [1,2,3,4].

HT alters cellular physiology both by modifying the physical properties of cellular components and by activating cellular stress responses. Its anticancer potential is based on multiple intracellular effects, including impaired DNA repair, cell cycle inhibition, modification of the tumor microenvironment (TME), immune response reactivation, altered vascularization, and improved oxygenation. In combination with chemotherapy and RT, HT further enhances ROS production, leading to oxidative damage of cellular macromolecules and cytoskeletal disorganization, energy depletion and disrupted calcium homeostasis [1,2,3,4,5].

Moreover, HT can support antitumor immunity, increase tumor oxygenation, and improve delivery of therapeutic agents, particularly within hypoxic or nutrient-deprived regions of tumors, which are typically less responsive to conventional treatments [6,7].

### 1.1. Cellular Changes

HT induces a broad spectrum of cellular alterations that disrupt homeostasis [4,8]. One of the most critical events is protein denaturation and aggregation, which leads to cell cycle arrest, inhibition of protein synthesis, and impaired DNA repair mechanisms [9,10]. Other cellular effects include suppression of DNA synthesis, transcription, RNA processing, and translation [4,8,9,10], accompanied by increased degradation of misfolded proteins through proteosomal and lysosomal pathways.

HT also alters the cytoskeleton, membrane permeability, and cellular metabolism, leading to reduced ATP levels and increased intracellular concentrations of ions such as Na^+^, H^+^, and Ca^2+^. Together, these changes promote cell death signaling. In the TME, increased membrane permeability and protein/fluid accumulation elevate interstitial fluid pressure (IFP). HT reduces IFP, enhances lymphatic drainage, and improves blood perfusion, thus facilitating the delivery of drugs or nanoparticles (NPs) [8,9,10,11,12].

Proteins are the main targets of HT at clinically relevant temperatures (39–45 °C). Heat exposure triggers diverse post-translational modifications, including glycosylation, acetylation, and ubiquitination, which impair protein stability and function [8,9,10,11,12,13]. Several studies have shown that HT leads to DNA fragmentation and double-strand breaks, likely due to impaired repair mechanisms. However, nuclear protein damage, rather than direct DNA damage, appears to be the key event. These proteins are highly sensitive to elevated temperatures and tend to aggregate, contributing to the inhibition of transcription and DNA replication.

Elevated temperatures also accelerate cellular metabolism, indirectly promoting oxidative stress. Both lethal (>42 °C) and sublethal ~40 °C) temperature exposure increase the ROS levels [13]. Mitochondrial respiratory chain dysfunction is a primary source of ROS such as superoxide and hydrogen peroxide (H_2_O_2_), while enzymes such as NADPH oxidase and xanthine oxidase may also contribute to ROS production under heat stress [13,14]. The cytotoxic potential of ROS is enhanced at elevated temperatures. For instance, H_2_O_2_ becomes significantly more toxic between 41 °C and 43 °C compared to physiological conditions [15]. Hyperthermia additionally weakens antioxidant defense systems, such as the pentose phosphate pathway, which is essential for maintaining intracellular levels of reduced glutathione (GSH) [15]. ROS overproduction leads to oxidative damage of proteins, lipids, and nucleic acids. In tumors, HT-induced reductions in growth have been associated with increased lipid peroxidation [12]. HT also disrupts MMP [16], further contributing to cellular dysfunction and apoptosis.

Finally, heat-induced acceleration of metabolism can lead to acidosis in tumor tissues [8]. The major physiological and molecular consequences of hyperthermia are summarized in Table 1.

### 1.2. Hyperthermia and Physiological Changes

Oxygenation, pH, and blood circulation are critical factors of cellular and tissue sensitivity to HT. A growing body of evidence indicates that tumor cells are generally more susceptible to thermal injury than normal cells [14]. This differential sensitivity is partly attributed to variations in the expression of heat shock proteins (HSPs), which play key roles in cellular defense mechanisms against stressors, including heat. Sensitivity to heat also varies across the cell cycle, and cells in the S phase and mitosis are particularly vulnerable [17].

A major rationale for using HT in cancer therapy lies in the abnormal vasculature of tumors compared to normal tissues. Tumor blood vessels are irregular and inefficient, leading to uneven heating. Consequently, higher temperatures are often reached in tumor tissues than in surrounding healthy ones, where normal blood circulation promotes heat dissipation. HT also complements other treatment modalities by effectively targeting tumor cells that are resistant to chemotherapy and RT [13].

Cells located in hypoxic regions of tumors are often poorly responsive to chemotherapy due to limited drug delivery. Additionally, the cytotoxic activity of certain chemotherapeutics depends on oxygen availability for free radical generation. As many chemotherapies preferentially target proliferating cells, the reduced proliferation associated with hypoxia may further contribute to treatment resistance [18]. Hypoxic tumor cells also show increased resistance to RT.

In clinical settings, temperature distribution within tumors often varies. Some tumor regions may reach cytotoxic temperatures of 43–45 °C, while others remain in the 39–42 °C range. Deep-seated and large-volume tumors are particularly difficult to heat uniformly. Unlike healthy tissues, tumors are unable to effectively regulate blood flow at ≥42 °C, leading to diminished nutrient and oxygen supply, decreased interstitial pH, and eventual vascular collapse [19,20]. These changes sensitize tumor cells to heat. Cells in an acidic, hypoxic environment, typically found in tumor cores, are more sensitive to HT. Nutrient-deprived cells are also more vulnerable to heat, partly due to reduced ATP levels. Cells with impaired glucose delivery exhibit heightened susceptibility to HT, possibly due to compromised antioxidant defenses. This is linked to impaired function of the GSH redox cycle, which depends on glucose flux through the pentose phosphate pathway.

Interestingly, mild hyperthermia (39–42 °C) can have beneficial effects by improving tumor blood flow and oxygenation [20], thereby enhancing tumor responsiveness to radiation and certain chemotherapeutic agents. Temperatures in the range of 40–41 °C also stimulate various components of the immune system. Exposure of immune effector cells (macrophages, T-cells, and NK cells) to these temperatures promotes: (i) migration of immune cells to tumor sites, improving immune surveillance, (ii) upregulation of surface molecules involved in antigen presentation, (iii) release of cytokines and other inflammatory mediators (e.g., tumor necrosis factor alpha (TNF-α), interleukin (IL)-1, IL-6, IL-10, and IL-12), (iv) proliferation of immune cells, and (v) cytotoxicity against tumor cells (see Table 1).

### 1.3. Thermotolerance Induction and HSPs

Short-term exposure of cells to lethal temperatures (43–45 °C for 10–30 min) can induce thermotolerance, a protective phenomenon in which cells become more resistant to subsequent stress exposures [21]. This adaptive response, triggered by heat preconditioning, enhances cell survival during latter challenges, such as exposure to thermal shock, ROS, and environmental toxins, including heavy metals. Under sublethal stress, cells activate protective pathways that preserve essential biochemical processes such as DNA repair, protein folding, and the removal of damaged proteins, thus allowing normal function to resume once the stress is removed [22]. However, prolonged or severe stress typically results in apoptosis or necrosis.

Thermotolerance involves numerous biochemical and molecular changes, although the exact mechanisms remain incompletely understood. However, accumulation of HSPs is widely considered as the key hallmark of this response [23]. Clinically, repeated or fractionated HT treatments may inadvertently promote thermotolerance, especially when mild heat is applied in subsequent sessions.

Heat preconditioning activates the JAK-STAT signaling pathway, leading to the upregulation of HSP27, HSP70, and HSP105, along with the increased expression of antiapoptotic members of the Bcl-2 family. This response inhibits caspase activation and suppresses apoptosis mediated by the FasL death receptor. Conversely, heat-induced autophagy has been shown to promote tumor invasion and metastasis through tumor growth factor (TGF)-β2/Smad2-mediated epithelial–mesenchymal transition in xenograft mouse models.

As HSPs help tumors resist thermal injury, targeting HSPs may enhance tumor sensitivity to HT. For instance, inhibition of HSP90 and HSP70 increases susceptibility to heat-induced apoptosis, suggesting HSPs as promising therapeutic targets in overcoming acquired thermotolerance. Additionally, suppressing autophagy may help prevent tumor progression and metastasis [4,24].

### 1.4. HSPs

Protein activity depends on its three-dimensional conformation, which is stabilized primarily by intramolecular interactions, notably hydrogen bonds [23]. Disruption of these bonds under external stress conditions leads to protein misfolding and denaturation. HSPs constitute a conserved family of molecular chaperones present in both prokaryotic and eukaryotic cells. They participate in a wide range of essential cellular processes, including protein folding, degradation of misfolded proteins, modulation of signaling pathways, and regulation of immune responses. Through these actions, HSPs play a pivotal role in maintaining cellular homeostasis and promoting survival under stress conditions.

**Figure 1:**
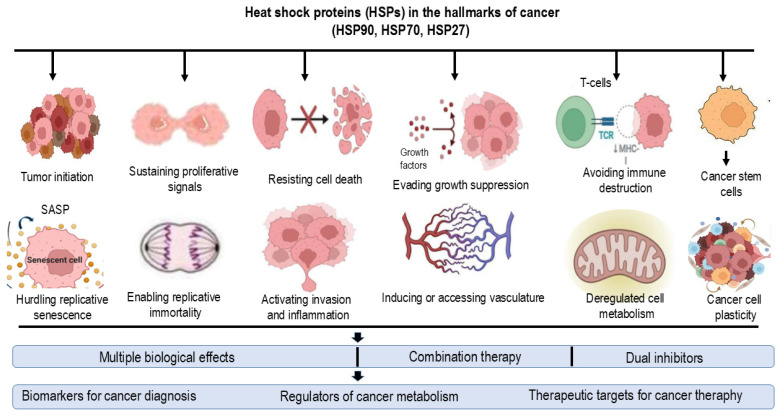
The role of heat shock proteins (HSPs) as markers for cancer diagnosis and progression, in the regulation of metabolism, and as targets in tumor therapy according to the hallmarks of cancer defined by Hanahan and Weinberg. *Abbreviations*: SASP—senescence-associated secretory phenotype.

HSPs are classified according to molecular weight (15–110 kDa) and functional specialization, and they are localized in the cytosol, mitochondria, ER, or nucleus. The most extensively studied mammalian HSPs have molecular weights of 60, 70, 90, and 110 kDa. These proteins are constitutively expressed under physiological conditions, but their levels are markedly upregulated in response to stress stimuli, including heat. Expression of HSPs is regulated by heat shock factor 1 (HSF1), a transcription factor that binds to heat shock elements (HSEs) in target gene promoters. Upon stress exposure, HSF1 undergoes Ras-dependent hyperphosphorylation mediated by mitogen-activated protein kinase (MAPK) pathways, including ERK1, JNK/SAPK, and p38 [23]. This induces trimerization, nuclear translocation, and DNA binding, thereby activating transcription of HSP genes. The heat shock response is evolutionary conserved from yeast to humans [23,24] and can be triggered by diverse stressors, including oxidative stress, heavy metals, infrared radiation, ethanol, toxins, viral infection, and bacterial pathogens. Elevated HSP expression is commonly observed in cancers and is associated with tumorigenesis, response to therapy and progression of the disease [23,24] (Figure 1).

The principal HSPs induced by heat stress include HSP90, HSP70, HSP60, and HSP27, all of which contribute to the development of thermotolerance. Accumulation of HSP70 correlates with enhanced thermotolerance in mammals, amphibians, insects, and fish, preventing protein aggregation and facilitating refolding of denatured proteins [23,24]. HSP110 functions similarly, cooperating with HSP70 and HSP40 in protein refolding. HSPs also confer protection against oxidative stress and radiation [23,24].

HSPs modulate apoptotic pathways at multiple levels [24]. HSP27, HSP60, HSP70, and HSP90 maintain mitochondrial integrity and regulate Fas receptor-mediated apoptosis. Specifically, HSP27 and HSP70 inhibit Fas-mediated apoptosis by preventing t-Bid translocation to mitochondria and blocking cytochrome c release, whereas HSP90 negatively regulates caspase-2 activation. Collectively, these HSPs interfere with both mitochondrial and post-mitochondrial apoptotic events, including apoptosome formation and caspase activation. Additionally, HSP70 and phosphorylated HSP27 mitigate oxidative stress-induced apoptosis [12].

Thermotolerance can be induced either by short exposure to lethal temperatures (42–45 °C for ~30 min) or prolonged exposure to sublethal HT (39–41.5 °C for 3–24 h) [24]. Mild HT (40 °C, 3–24 h) induces HSP27, HSP32, HSP60, HSP70, HSP90, and HSP110, providing protection to normal tissues during clinical HT. In vivo exposure of Balb/c mice to physiological HT (39.5–40 °C for 6 h) upregulated HSP70 and HSP110 in multiple tissues [26]. This mild thermotolerance generates an apoptosis-resistant phenotype, reducing both mitochondrial and Fas receptor-mediated apoptosis under subsequent moderate HT (42–43 °C) or oxidative stress (H_2_O_2_) [12,27]. This phenotype is associated with increased expression of HSPs and activity of antioxidant enzymes, including catalase, superoxide dismutase (SOD), and GSH, and reduced ROS accumulation [27].

In cancer, HSPs facilitate tumor cell survival, proliferation, and chemoresistance. HSP70 overexpression is observed in multiple malignancies and is associated with increased proliferation, poor differentiation, and reduced response to therapy [23,24]. HSP27 contributes to chemoresistance against agents such as doxorubicin and cisplatin (CP) [28,29]. The multifunctional roles of HSPs in cancer progression make them attractive therapeutic targets, and small-molecule inhibitors have been developed to selectively disrupt HSP70 and HSP90 function.

The HSP90 family consists of four members: (i) HSP90α (inducible) and HSP90β (constitutive), localized in the cytosol and nucleus; (ii) GRP94 in the ER; and (iii) TRAP1 (HSP75), predominantly mitochondrial with ER localization [30]. HSP90 supports oncogenic signaling, stabilizes mutated proteins, inhibits apoptosis, and promotes proliferation, migration, epithelial–mesenchymal transition (EMT), angiogenesis, and chemoresistance. HSP90 also regulates extracellular matrix remodeling via matrix metalloproteinase 2 (MMP2), matrix metalloproteinase 9 (MMP9), tissue plasminogen activator (tPA), lysyl oxidase like 2 (LOXL2), and fibronectin, influencing tumor stiffness and aggressiveness. HSP90 also modulates oncogenic proteins, including p53, Bcr-Abl, Akt, Her2, Cdk4, Cdk6, Raf, and Src. HSP90-TLR4 interactions activate epidermal growth factor receptor (EGFR) signaling, further promoting malignancy, EMT, invasion, and chemoresistance via Akt, Src, ERK, focal adhesion kinase (FAK), vascular endothelial growth factor (VEGF), and STAT pathways [30].

HSP27 regulates the cell cycle by suppressing inhibitory proteins (E2F-4 and p130) and promoting expression of cell cycle regulators (CCNA2, CCNB1, CCNB2, CDC25C, CDC3A, and CDK1) [31]. HSPs also play a critical role in cancer stem cell (CSC) maintenance, supporting adaptation to nutrient deprivation, hypoxia, and inflammation. CSCs contribute to tumor heterogeneity, self-renewal, differentiation, metastatic potential, therapy resistance, enhanced DNA repair capacity, and long-term tumor repopulation [32]. CSCs can arise either via oncogenic or epigenetic modifications of normal stem cells, or through dedifferentiation of mature cancer cells [33]. The TME, including immune, stromal, endothelial, and epithelial components, regulates CSC survival through HSP upregulation, DNA repair activation, antiapoptotic signaling, drug efflux, and ROS detoxification [34].

In summary, HSPs enable tumor cells to survive genotoxic and oxidative stress, including chemotherapy and RT, by modulating signaling pathways, promoting proliferation, inhibiting apoptosis, supporting invasion and metastasis, and regulating DNA repair. Ultimately, this multifunctional support facilitates tumor adaptation and survival under adverse conditions [35].

## 2. Hyperthermia in Cancer Therapy

Temperatures of 42.5 °C and above can destroy tumor cells. In in vitro studies, no intrinsic differences in thermal sensitivity have been observed between normal and tumor cells [1,2,3,4,5]. However, in in vivo conditions, tumor cells exhibit selective sensitivity at higher temperatures. This is largely due to the disorganized vasculature of solid tumors, which generates regions of hypoxia and low pH compared with normal tissues, making tumor cells more susceptible to the cytotoxic effects of HT. Consequently, hyperthermia can induce direct tumor cell death, with preferential destruction occurring in hypoxic, acidic regions of solid tumors.

**Figure 2:**
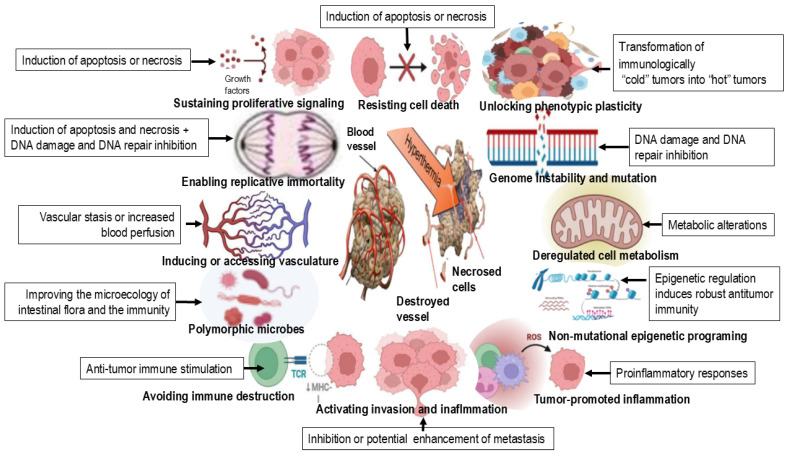
Main effects and mechanisms of hyperthermia on tumors. Elevated temperatures due to heat shock disrupt DNA repair mechanisms, resulting in replication errors, cell cycle arrest, and apoptosis. In addition, hyperthermia decreases telomerase activity through direct enzyme inactivation and modulates both the function and composition of the intestinal microbiota and the immune response.

In clinical practice, hyperthermia is most effective when combined with RT and/or chemotherapy. Local heating of tumors to approximately 42 °C for 60 min enhances cancer treatment outcomes by directly killing tumor cells and sensitizing them to radiation and chemotherapeutic agents. This synergistic effect arises from several mechanisms: (i) inhibition of DNA double-strand break repair pathways (HT impairs homologous recombination and non-homologous end joining), leading to the lethal accumulation of DNA damage [8,9,10]; (ii) increased tumor cell membrane permeability, which enhances intracellular uptake of chemotherapeutic drugs; and (iii) improved tumor oxygenation, which mitigates hypoxia-induced radioresistance [18,20] (see Figure 2 and Table 2).

### 2.1. The TME as a Target of Hyperthermia

The TME includes cellular and molecular components that together shape conditions favorable for tumor development, progression and spread. Physiological factors such as oxygen availability and the pH are also important in defining the local TME [36,37]. By secreting various chemokines and proinflammatory substances, tumor cells can induce surrounding stromal cells to establish a more reactive phenotype that supports tumor progression.

Tumor-associated stromal cells (TASCs) express surface proteins and secrete cytokines that attract immunosuppressive inflammatory cells, creating an environment conducive to tumor growth [38,39,40]. Besides TASCs, the TME contains cells of specific and nonspecific immunity, including T- and B-lymphocytes, dendritic cells, macrophages, polymorphonuclear leukocytes (neutrophils) and NK cells (Table 2).

Cytotoxic T-lymphocytes (CTLs) are the most effective antitumor cells that recognize and destroy tumor cells expressing specific antigens. Helper T1 (Th1) lymphocytes support them by secreting cytokines such as interferon (IFN)-γ and IL-2, while helper T2 (Th2) lymphocytes support activity of B-cells by secreting various interleukins (IL-4, IL-5, and IL-13). Th17-lymphocytes maintain inflammatory conditions by secreting interleukins such as IL-17A, IL-17F, IL-21 and IL-22, facilitating tumor progression. Regulatory T-lymphocytes have an immunosuppressive effect through contact-dependent mechanisms or via cytokine secretion (IL-10 and TGF-β) [36,37,38,39,40].

NK cells induce apoptotic death through death receptors and secrete cytotoxic substances (perforin and granzyme). Tumors employ various strategies to evade NK cell cytotoxicity, such as collagen or platelet coating to prevent contact with “death receptors” [36,37,38,39]. However, tumor-associated natural killer cells (TANKs) within the TME can change cytokine production and stimulate the secretion of proangiogenic factors [38].

Neutrophils initially contribute to genomic instability and mutagenesis through secretion of cytotoxic substances. During tumor progression, they gradually acquire an immunosuppressive phenotype and secrete substances without detrimental effects on tumor cells. Two distinct tumor-associated neutrophil (TAN) phenotypes are distinguished, antitumor N1 and protumor N2 [36,39]. N1 neutrophils secrete cytotoxic substances (ROS and nitric oxide (NO)), produce extracellular matrix proteases (neutrophil elastases and matrix metalloproteases) and form neutrophils extracellular traps (NETs) [36,39,41]. In contrast, TGF-β-promoted polarization toward the immunosuppressive N2 phenotype is characterized by reduced secretion of cytotoxic substances and diminished antitumor activity. N2 neutrophils secrete protumor and inflammatory mediators that stimulate angiogenesis and tumor progression, such as IL-6 and prostaglandin E2, and create conditions that enable invasion. However, NETs can degrade proinflammatory cytokines and chemokines like IL-12 and IL-23. In addition, neutrophil-derived chemokines may affect differentiation of macrophages into protumor cells [36,39,41].

The functions of mast cells are context-dependent, ranging from antitumor effects to the promotion of angiogenesis and tumor stroma shaping. Furthermore, they may promote eosinophil infiltration and tumor cell apoptosis [36].

Tumor-associated macrophages (TAMs) include M1 macrophages with antitumor activity, characterized by the secretion of inflammatory cytokines (TNF-α, CXCL10, and IL-1β) and antigen presentation [36,37,38,39,40,41], and M2 macrophages with protumor and anti-inflammatory activity. M2 macrophages are polarized by tumor-derived signals and hypoxic conditions. They promote tumor cell proliferation, angiogenesis and epithelial–mesenchymal transition (EMT), inhibit lymphocyte activity by secreting cytokines (IL-10 and prostaglandin E2), and contribute to tissue remodeling by secreting various proteases (MMP9, MT1-MMP, and MMP2) [36,37,38,39,40,41].

Myeloid-derived suppressor cells (MDSCs) are immature myeloid cells. They stimulate tumor growth and inhibit antitumor immune responses. MDSCs suppress immune function through the activity of arginase 1 and inducible nitric oxide synthase (iNOS), leading to the production of NO and ROS. Tumor-derived factors, including IL-10, VEGF, and granulocyte–macrophage colony-stimulating factor (GM-CSF), promote the development and accumulation of MDSCs within the TME, while blocking the maturation of dendritic cells and formation of specialized lymphocytes [36,37,38,39,40,41]. Dendritic cells patrolling the TME encounter various cytokines and immunosuppressive factors (VEGF, IL-10, TGF-β, and prostaglandin E2) that inhibit their maturation into functional immune cells. As a result, dendritic cells acquire a tolerant phenotype, losing the ability to activate Th1, Th2 and Treg lymphocytes, which ultimately limits the efficacy of antitumor immune responses [36,37,38,39,40,41].

The tumor stroma consists of fibroblasts, adipocytes, vascular endothelial cells, perivascular cells, and mesenchymal stem cells [36,37,38,39,40,41]. Endothelial cells play a key role in tumor protection and angiogenesis, facilitating nutrient supply and metastatic spread [36,37,38,39,40,41].

Mesenchymal cells help with communication between tumor and stromal cells and can differentiate into pericytes or fibroblasts. In the TME, cancer-associated fibroblasts (CAFs) support tumor proliferation, angiogenesis, and tumor cell invasion into the vasculature [36,37,38,39,40,41].

Cancer-associated adipocytes (CAAs), influenced by tumor cells, adopt a reactive phenotype and secrete interleukins (IL-6, IL-8, and IL-1β) that attract immune and stromal cells. CAAs also remodel the extracellular matrix and can serve as an energy source through lipolysis [38].

Hyperthermia (40–43 °C) targets the tumor microenvironment (TME) by reversing its immunosuppressive, hypoxic and acidic nature, acting as a potential adjuvant (see Table 2) to improve therapeutic outcomes.

### 2.2. Key Conditions for Tumor Escape in the Hyperthermal Microenvironment

The altered metabolism of tumor cells, particularly the byproducts of the glycolytic pathway, contributes to the formation of hypoxic and acidic TMEs. Rapid proliferation requires a substantial supply of energy, leading to nutrient depletion in the TME and increased susceptibility to oxidative stress.

These unfavorable conditions impose strong selective pressure, allowing for the survival of genetically unstable, invasive cells capable of metabolic adaptation. Tumor cells often shift from oxidative phosphorylation to aerobic glycolysis (the Warburg effect), enabling survival under hypoxic conditions [13].

Inflammatory cells such as macrophages and granulocytes also rely on glycolysis in low-oxygen environments, further exacerbating hypoxia by producing ROS. Due to the high rate of apoptosis in tumor cells induced by these cytotoxic substances, phagocytes are exposed to various activation and polarization signals that promote their polarization toward protumorigenic and immunosuppressive phenotypes. Excessive ROS levels may also compromise lymphocyte viability [13,39,41].

CTLs undergo metabolic reprogramming upon activation, switching to glycolysis, whereas memory and regulatory T-cells depend on oxidative phosphorylation and fatty acid oxidation, making them less viable under hypoxic conditions. Nutrient depletion in TME, particularly reduced glucose levels due to competition with tumor cells, results in metabolic starvation of T-lymphocytes [42].

As mentioned previously, the glycolytic switch in tumor cells leads to lactate accumulation and extracellular acidification. To maintain intracellular pH homeostasis, tumor cells activate proton pumps that expel hydrogen ions, further acidifying the TME. This low-pH environment suppresses T-cell function, contributing to immune evasion and promoting mutations in signaling pathways that support tumor progression [13,42,43].

Despite infiltration by innate and adaptive immune cells, their ability to control tumor growth is often compromised through the manipulation of immune checkpoints, including the downregulation of activating immune receptors and upregulation of inhibitory receptors [13,24,36,37,38,39,40,41,42,43].

Tumor cells evade immune surveillance via several strategies, including impaired antigen presentation and the activation of immune checkpoints, which inhibit T-cell activation. The presence of immunosuppressive cells, such as regulatory T-cells, TAMs, and MDSCs, as well as inappropriate chemokine signaling, can also inhibit T-cell cytotoxicity. Expression of inhibitory receptors, such as PD-1, CTLA-4, LAG-3, TIM-3, TIGIT, and BTLA, further suppresses immune responses [13,24,36,37,38,39,40,41,42,43].

To counteract this immunosuppression, several forms of immunotherapy have been developed. These include immune checkpoint blockade therapy (ICBT), adoptive cell therapy, cytokine therapy, cancer vaccines and natural products (12). ICBT targeting the CTLA-4 and PD-1/PD-L1 pathways has shown considerable success in treating advanced melanoma, non-small-cell lung cancer (NSCLC), renal cell carcinoma (RCC), Hodgkin’s lymphoma, and other tumors [13,24,39,44,45].

### 2.3. Clinical Methods of Hyperthermia Application

HT can be classified into three categories based on the method of application: local, regional and whole-body HT (see Table 3) [46,47]. Local HT involves the increase in temperature of specific tumors using microwaves, radio waves or ultrasound. It is commonly applied to tumors on the skin or natural body surfaces, such as metastases in cervical lymph nodes and skin tumors. Regional HT is achieved by heating organs or limbs, often through perfusion with heated liquid, e.g., intraperitoneal HT, and is frequently combined with chemotherapy and radiofrequency in deep-seated tumors. Whole-body HT is primarily used for the treatment of metastatic cancers. Depending on the heating medium, it can be divided into microwave heating therapy, infrared heating therapy, magnetic heating therapy, and photothermic therapy, among others. Due to the limitations of current techniques, a major challenge in HT therapy is accurate positioning and temperature control of the treatment area. However, advances in nanotechnology have expanded its application, especially in photothermal therapy (PTT) or magnetic nanoparticle-based “nanothermotherapy” [48,49]. Magnetic nanoparticles generate heat only when suitable magnetic field is applied across the tissue, allowing for localized and well-controlled heating.

In recent years, HT has been combined with immunotherapy, as tumors often evade immune response due to the presence of a large number of immunosuppressive cells in the tumor microenvironment. For example, combining anti-PD-1/PD-L1 immunotherapy combined with RT and HT can enhance the antitumor effect [13,24,50,51,52,53]. There are still many unknown aspects of HT. Deeper mechanistic insights could improve HT-based cancer therapy not only by overcoming thermoresistance but also by guiding the development of new thermosensitizers and optimized thermotherapy protocols. An important aim of this review is to provide a concise overview of mechanism, methodologies and clinical applications of hyperthermal chemotherapy, particularly in combination with natural products.

### 2.4. Cytotoxicity of Hyperthermia in Cancer Therapy

The selective cytotoxicity of HT towards tumor cells is related to the physiological differences between tumor and normal tissues. The vascular network of solid tumors is unevenly developed, resulting in regions of hypoxia and low pH. These conditions make malignant cells more sensitive to HT than normal cells. In addition, impaired microcirculation in tumor tissues reduces heat dissipation, nutrient supply, and oxygen availability, further enhancing their susceptibility to high temperatures [1,4,13,18,24,53,54,55].

Vascular abnormalities contribute to hypoxic environment within the tumor. Mild HT (37 °C to 42 °C) can induce local vasodilation and increase vascular perfusion, thereby improving tumor oxygenation. This may reduce inflammation, promote deep tissue hyperemia, decrease nerve excitability, and consequently alleviate pain. In contrast, at temperatures above 42 °C, tumor vasculature becomes directly damaged due to increased vascular permeability. This results in fluid and protein accumulation in the microenvironment, elevating interstitial fluid pressure, consequently leading to vessel compression and further reduction in vascular perfusion. Furthermore, the mechanism responsible for vascular injury is activated, and tumor growth and proliferation are inhibited during the heating process. On the contrary, HT enhances microcirculation in normal cells. At 42–44 °C, blood flow in muscles and skin can increase by 5- to 10-fold, while blood flow in tumor tissue shows only minimal changes under same conditions [56,57]. HT also induces structural and functional changes in cells: temperatures of 40–45 °C typically trigger apoptosis, while temperatures above 45 °C lead to necrotic cell death. For example, exposing cells to moderate HT, 43 °C for 1 h, can induce apoptosis in various cell cultures. The intensity of apoptosis depends on the cell type, temperature, recovery time, cell cycle phase and protein synthesis [58,59].

HT increases cellular permeability, enhancing passive transport. It also disrupts microtubules, impairing spindle formation during mitosis and increasing thermosensitivity of dividing cells. Mitochondrial swelling, rupture and loss of function further contribute to cell death. Furthermore, HT inhibits protein synthesis on cytosolic polyribosomes, and damages lysosomal membranes, releasing hydrolytic enzymes that promote cell death during interphase. Tumor lysosomes are generally more thermolabile than those in normal cells. Nuclear damage induced by heat inhibits DNA synthesis and repair, amplifying cytotoxic effects.

The basic cellular response to HT is activation of genes encoding HSPs. They are regulated by physiological processes such as cell cycle, cell proliferation and differentiation. However, stressors such as HT, ROS, chemotherapeutics, ischemia, fever, inflammation, infection and tumors can all induce HSP overexpression [13,60]. HSPs protect tumor cells from apoptosis, contributing to resistance to chemotherapy and HT [60,61]. They regulate protein synthesis, assist in protein transport across cell membranes, and prevent formation of non-functional aggregates by adhering to the hydrophobic sites of cellular proteins. This cytoprotective function enables cells to survive and repair heat-induced damage. However, prolonged HT or exposure to additional toxic factors can overwhelm HSP capacity, leading to apoptosis [60,61].

Inhibition of genes encoding HSPs increases thermosensitivity of cells. Furthermore, HT stimulates pro-oxidative reactions, leading to elevated production of ROS, oxidative stress and cell apoptosis [13,60,61]. Therapeutic HT increases HSP expression on the surface of both normal and tumor cells. However, following recovery at 37 °C, HSP expression is present predominantly in tumor cells. Cells expressing HSPs are more susceptible to cytotoxic activity mediated by NK cells. Additionally, HSPs released during cell necrosis stimulate macrophages and dendritic cells to secrete cytokines and activate antigen-presenting cells, thereby enhancing the immune response against tumor cells [13,21,23,60,61]. Lipid peroxidation, driven by ROS, further contributes to apoptosis and strengthen the antitumor effect of HT. Due to their reduced antioxidative capacity, tumor cells are more sensitive to ROS and other reactive species [13].

**Figure 3:**
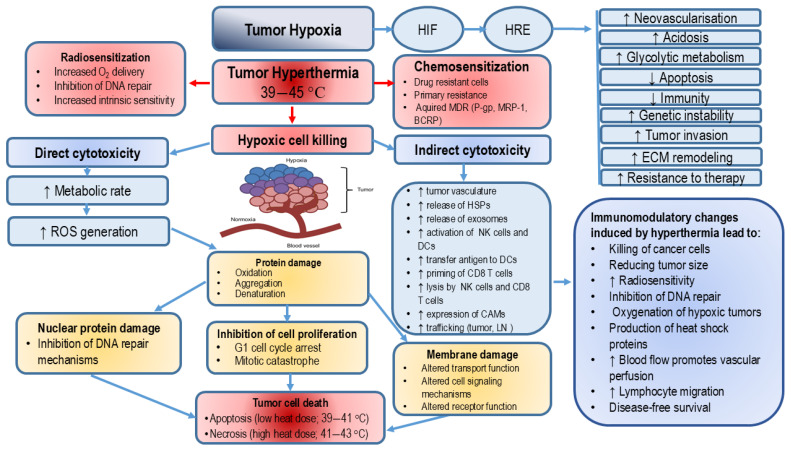
Direct and indirect cytotoxic effects of hyperthermia in cancer therapy. Tumor hypoxia promotes cancer progression, metastasis, and therapy resistance through activation of hypoxia-inducible factor 1 (HIF-1). HIF-1 binds hypoxia-responsive elements (HREs) in DNA, inducing genes involved in angiogenesis, metastasis, metabolic reprogramming (e.g., increased glycolysis), cell survival, immune evasion, inflammation, and proliferation. Hyperthermia alters tumor physiology and improves chemo- and radiotherapy efficacy. It promotes tumor perfusion and vascularization, reduces oxygen consumption, and decreases tumor hypoxia in a temperature- and time-dependent manner. These modifications in tumor physiology can positively influence the intratumoral accumulation and distribution of small-molecule chemotherapeutics and nanomedicines, as well as increase radiosensitivity. Hyperthermia can also induce direct DNA damage in cancer cells through several mechanisms, including the generation of reactive oxygen species (ROS) and disruption of DNA repair pathways, ultimately leading to genomic instability and cell death. In addition, hyperthermia downregulates proangiogenic and pro-invasive factors, such as TGF-β1, VEGF, and MMP-2/9, thereby contributing to reduced tumor progression. The indirect cytotoxic effects of hyperthermia are based on its ability to potentiate immune responses and to elicit both nonspecific and specific antitumor activities. ↑ increase; ↓ decrease. *Abbreviations*: CAMs, cell adhesion molecules; DCs, dendritic cells; HSPs, heat shock proteins; LN, lymph node; MDR, multidrug resistance; NK cells, natural killer cells; P-gp, P-glycoprotein; ROS, reactive oxygen species; TGF-β1, transforming growth factor beta 1; VEGF, vascular endothelial growth factor.

HT induces a variety of cellular changes, leading to mitotic catastrophe, permanent arrest in the G1 phase, and loss of reproductive potential. Depending on the cell type, temperature and duration of exposure, cells may die by processes such as apoptosis and/or necrosis [62]. HT also sensitizes cells to other cytotoxic agents, such as radiation. It has been shown that HT causes centrosomal dysfunction and mitotic catastrophe [63], which underlies its thermal radiosensitization effects. HT also promotes chromatin condensation and apoptotic DNA fragmentation, leading to apoptosis in multiple cell types, including HeLa cells, T-lymphocytes, HL-60 leukemia cells, and mouse embryonic fibroblasts [64]. HT triggers apoptosis in various tumors, including breast cancer, skin cancer, osteosarcoma, melanoma, skin cancer, colon cancer, head and neck cancer, esophageal cancer, glioblastoma multiforme, cervical cancer, bladder cancer and lung cancer, primarily through changes in the ER [65]. In mice exposed to whole-body hyperthermia (41.5 °C for 2 h), the extent and kinetics of apoptosis differed between tumor types (fibrosarcoma versus colon carcinoma) [66]. More importantly, apoptosis induction was more prominent in tumor cells compared to normal cells. Most studies investigating the mechanisms of heat shock-induced cytotoxicity concluded that apoptosis is the primary mode of cell death, highlighting the proapoptotic effects of HT as potentially valuable strategy in cancer therapy (see Figure 3).

### 2.5. Heat Stress, ROS Production and Apoptosis

Dysfunctional apoptotic processes are a hallmark of tumor cells. HT induces cell death through several mechanisms. Firstly, elevated temperatures cause protein unfolding and aggregation that can exceed the capacity of HSPs, leading to the accumulation of unfolded proteins and ER stress and consequently cell death through the activation of caspases 12 or 4. Secondly, HT induces various forms of DNA damage (base modification, single-strand breaks and replication inhibition). These events activate the ATM-Chk2 or ATR-Chk1 pathways, resulting in cell cycle arrest and subsequent apoptosis. Thirdly, HT triggers mitochondrial (intrinsic) and extrinsic apoptotic pathways. It alters the permeability of the outer mitochondrial membrane, promoting the release of cytochrome c and activation of caspase-dependent apoptosis, as well as apoptosis-inducing factor (AIF) or second mitochondria-derived activator of caspases (Smac)/Diablo, leading to the formation of apoptosome. Extrinsic signaling pathways are activated via cell surface death receptors, including Fas, TRAIL and TNF-α signaling. Other important factors in the induction of HT-induced apoptosis are oxidative stress, p53, Bax/Bcl-2, and increased intracellular Ca^2+^ levels [13,67,68].

Several studies have shown that hyperthermic intraperitoneal chemotherapy (HIPEC), a type of locoregional chemotherapy, may prolong the survival of patients with peritoneal carcinomatosis. This effect is mediated through the induction of apoptosis, angiogenesis inhibition, and direct cytotoxic effect via protein denaturation. HIPEC-induced cancer cell death is associated not only with elevated temperatures and chemotherapy drugs, but also with oxidative stress induced by HT [12,67,68,69].

HT-induced mitochondrial dysfunction plays a key role in cell death. Increased mitochondrial membrane permeability, excessive generation of ROS, and disruption of the respiratory chain impair ATP production, limiting cellular energy supply and increasing susceptibility to stress.

Heat stress increases the level of mitochondrial superoxide anion (O_2_^−•^). O_2_^−•^ is the primary byproduct of the one-electron reduction in molecular oxygen. This reaction occurs at specific points of the electron transport chain in mitochondria, which are an important source of O_2_^−•^ during HT. The formation of O_2_^−•^ is also closely related to the monovalent reduction in oxygen by mitochondrial semiquinone intermediates. Semiquinone radicals have been directly observed in the portal blood of hyperthermic rats [70,71,72,73,74]. It is possible that thermal denaturation of redox flavin-containing proteins may further increase their reduction by oxygen, thereby increasing O_2_^−•^ formation. In addition, heat stress decreases superoxide dismutase 1 (SOD-1) mRNA levels, SOD levels in the cytoplasm, and enzyme activity, altogether resulting in increased ROS production [67].

Heat stress also leads to overproduction of transition metal ions, which can donate electrons to oxygen, forming additional O_2_^−•^ [71,72,73,74]. Specifically, HT increases iron release from ferritin, and the extent of iron release correlates positively with levels of the O-type form of xanthine oxidase (XO), thereby enhancing the rate of O_2_^−•^ production [75]. O_2_^−•^ is the precursor of most ROS and plays a central role in oxidative chain reactions. Although highly reactive, it does not diffuse easily across the cell membrane. O_2_^−•^ is converted into H_2_O_2_, either spontaneously or through the SOD-catalyzed reaction. It can also react with NO to form peroxynitrite (ONOO^−^), a very powerful oxidant [67,76,77,78].

H_2_O_2_ is another key mitochondrial ROS, generated primarily through the dismutation of O_2_^−•^ by MnSOD. Heat stress increases H_2_O_2_ levels and also enhances formation of hydroxyl radicals (OH•), whose increased production reflects increased formation of O_2_^−•^ [67,76,77,78]. As HT enhances the release of redox-active transition metal ions, electron transfer to oxygen promotes H_2_O_2_ accumulation. H_2_O_2_ is further reduced to extremely reactive hydroxyl radical via the Fenton reaction. Due to its small size and relatively low reactivity compared with other radicals, H_2_O_2_ freely diffuses across cell membranes and can exert toxic effects far away from the site of its production. In contrast, OH• has an extremely short half-life and reacts almost immediately with molecules in close proximity [67,76,77,78].

H_2_O_2_ is also capable of inducing reversible covalent modifications of thiol groups in active and allosteric sites of proteins containing deprotonated cysteine (Cys) residues, thereby altering their activity and function. Protein thiols can undergo further two-electron oxidation with H_2_O_2_, leading to the formation of sulfinic (R–SO_2_H) or sulfonic acids (R–SO_3_H). Different types of proteins are susceptible to H_2_O_2_-mediated thiol modifications, including phosphatases, transcription factors, ion channels, antioxidant and metabolic enzymes, structural proteins and protein kinases [76].

On the other hand, HT increases the production of NO, a vasodilator generated through the breakdown of arginine to citrulline, in a reaction catalyzed by a family of nicotinamide adenine dinucleotide phosphate (NADPH)-dependent enzymes knowns as nitric oxide synthases (NOSs) [79,80]. Severe HT increases NOS levels and stimulates NO release [80]. Within mitochondria, NO bounds to heme groups in cytochromes (particularly cytochrome oxidase) and inhibits the respiration process. This inhibition of respiration stimulates formation of O_2_^−•^. Peroxynitrite (ONOO^−^), formed in the reaction of NO with O_2_^−•^, is a highly deleterious species with a broad spectrum of cellular targets. ONOO^−^ modifies proteins via tyrosine nitration, dityrosine formation, and oxidation of tryptophan and Cys residues. The main mitochondrial targets of ONOO^−^ include complexes I, II, IV and V of the electron transport chain, aconitase, creatine kinase, SOD, mitochondrial membranes and mitochondrial DNA (mtDNA) [76]. Beside mitochondria, ROS are also generated by NADPH oxidases, which catalyze the conversion of NADPH to NADP^+^. HT increases the NADP+/NADPH ratio without affecting NADPH subunits and upregulates NADPH oxidase 1 (NOX1) mRNA expression [81]. According to Moon et al. [81], HT activates NADPH oxidases via NOX1 upregulation. NOX-mediated ROS production via the ERK signaling pathway leads to the activation of hypoxia-inducible factor-1 (HIF-1). Therefore, heat stress stimulates HIF-1 through ERK-NADPH oxidase-mediated ROS production, enhancing tumor oxygenation by upregulating HIF-1 target genes involved in tumor perfusion, vascularization and metabolism. These findings suggest that HIF-1 may play a beneficial role in radiosensitizing tumors following HT [81].

### 2.6. The Role of Hypoxia-Inducible Factor-1 in Hyperthermia-Induced Tumor Reoxygenation and Therapy Resistance

HIF-1 promotes tumor growth by stimulating: (i) angiogenesis via VEGF and stromal cell-derived factor-1 (SDF-1); (ii) metabolic adaptation through the regulation of glucose transporters GLUT-1 and GLUT-3 and glycolytic enzymes; (iii) regulation of cell apoptosis and cell survival through BNIP-3, p53, TGF-β and basic fibroblast growth factor (bFGF). HIF-1α also contributes to metastasis by altering cell adhesion and motility by regulating EMT and expression of E-cadherin, zinc finger E-box-binding homeobox proteins 1 and 2 (ZEB 1/2) and transcription factor 3 (TCF3). In addition, it facilitates migration and invasion via C-X-C chemokine receptor type 4 (CXCR4), carbonic anhydrase IX (CAIX), lysil oxidase (LOX), MMP2 and MMP9.

Excessive expression of HIF-1α and HIF-2α subunits enhances blood vessel formation, tumor malignancy, metastasis and resistance to therapy. Activation of the HIF pathway triggers transcription of more than 100 genes associated with angiogenesis and metastasis, such as VEGF, SDF-1, angiopoietin-1 (Ang-1), placental growth factor (PlGF), platelet-derived growth factor B (PDGF-B), stem cell factor (SCF), vascular endothelial cadherin (VE-cadherin), ephrin-B, CD31, semaphorin 4D (sema4D), plexinB1, integrins, MMPs, and nodal and endothelial VEGF receptors (VEGFR-1 and VEGFR-2) [82]. Members of the HIF family play distinct roles in angiogenesis: HIF-1α promotes the sprouting of new blood vessels, while HIF-2α contributes to blood vessel maintenance. HIF-1 is primarily involved in the acute hypoxic response (<24 h), while HIF-2 mediates the chronic response (>24 h). The interplay between free radicals and HIF signaling should also be emphasized. NO exhibits both pro-oxidative and antioxidative properties. In reaction with O_2_^−•^, NO generates highly toxic peroxynitrite that stimulate lipid peroxidation [83]. On the contrary, peroxynitrite can react with lipophilic peroxyl radicals (ROO•) forming the more stable ROONO that terminates lipid peroxidation.

HT promotes tumor perfusion/vascularization, reduces oxygen consumption, and alleviates tumor hypoxia. By stimulating tumor angiogenesis and increasing blood flow, HT induces a cascade of changes in the TME. Even in the presence of enhanced oxygen delivery, reduced oxygen consumption has been shown to facilitate tumor reoxygenation after HT [84]. The mechanism of reduced oxygen consumption is based on increased accumulation of lactate in tumor tissue [84] and impaired mitochondrial function in tumor cells following HT [59]. Specifically, the metabolic shift from oxidative phosphorylation to glycolysis reduces tumor oxygen consumption and increases lactate levels. Hypoxic tumors are typically resistant to RT and chemotherapy due to increased DNA damage repair and poor drug delivery and diffusion [85]. In contrast, combined HT treatment exerts a synergistic effect, improving therapeutic outcomes by overcoming these limitations through tumor reoxygenation. Therefore, the administration of mild HT prior to radiation and chemotherapy can significantly enhance treatment efficacy. Notably, HIF-1 activity may have both beneficial and detrimental effects. While HT-induced upregulation of HIF-1 contributes to tumor reoxygenation, its role in tumor progression and resistance to therapy makes HIF-1 a promising therapeutic target.

Mild hyperthermic therapy increases blood flow, enhances tumor perfusion, and increases oxygenation, thereby improving the effectiveness of chemotherapy, RT and the antitumor immune response. At higher temperatures, HT inhibits DNA repair mechanisms leading to an increase in replication errors, cell cycle arrest, and induction of apoptosis. On the other hand, thermal ablation induces necrotic cell death, releasing a large number of tumor antigens, which enhance the antigen-presenting activity of macrophages and stimulate the immune response (see Table 4).

### 2.7. Cancer Stem Cells as a Target of Hyperthermia

The eradication of all malignant cells is the ultimate—yet challenging—goal of cancer treatment. Within tumors, certain cells can evade therapy or become resistant to clinically available treatments. CSCs, in particular, are highly therapy-resistant and implicated in disease recurrence. They play a crucial role in tumor initiation, progression and metastasis [86]. CSCs are characterized by several defining properties, including heterogeneity, self-renewal, unlimited proliferation, differentiation, plasticity, enhanced DNA repair capacity, metastatic potential, resistance to therapy, and ability to migrate and re-establish entire tissues, organs, or tumors. They can expand through cell proliferation or originate from non-CSCs via dedifferentiation. Microenvironmental factors such as low pH, hypoxia and inflammation contribute to CSC plasticity. Anticancer treatments, including RT and chemotherapy, can induce the dedifferentiation of non-CSCs into CSCs.

Heterogeneity within CSC cells arises from abnormal differentiation and accumulation of different mutations. In animal models, CSCs show 10 to 50 times higher tumorigenicity compared to other tumor cells. CSCs are characterized by reduced or even arrested cell cycle progression and increased DNA repair capacity, limiting the success of conventional therapies. CSCs are associated with tumor aggressiveness and therapy resistance due to the maintenance of a quiescent state, as well as mechanisms such as EMT, self-renewal, interactions with tumor environment, dormancy, epigenetic modifications, multidrug resistance (MDR), and increased aldehyde dehydrogenase (ALDH) activity.

HT represents a promising approach for selective targeting of CSCs in solid tumors. Its advantages include: (i) targeting hypoxic, nutrient-deficient tumor areas where CSCs reside, i.e., areas where ionizing radiation and chemotherapy are least effective; (ii) affecting factors essential for tumor growth and survival, especially those affecting the tumor microenvironment, immune response, vascularization and oxygen supply; (iii) interference with multiple DNA pathways, which are often upregulated in CSCs, preventing the repair of damaged DNA and promoting cell death. Therefore, HT represents a promising strategy for the elimination of therapy-resistant cells and those that evade conventional approaches [83,87].

The combined effects of chemotherapy or RT with HT on CSCs include multiple mechanisms: stimulation of blood flow and reoxygenation of hypoxic tumor areas, increased vascular permeability, activation of the immune response, and inhibition of DNA repair. Conventional tumor therapies, when combined with HT, directly sensitize CSCs by promoting blood vessel leakage, which enhances the delivery of chemotherapeutics into deeper tumor regions. Reoxygenation increases tumor oxygen, thus enhancing the sensitivity of CSCs to RT. In addition, HT increases the number of DNA breaks and impairs DNA repair, leading to the accumulation of DNA damage. Simultaneously, activation of immune system cells by HSPs acting as danger signal can disrupt the CSC microenvironment [88,89].

### 2.8. Hyperthermia and Immunity

Hyperthermia plays a key role in strengthening the body’s immune response and inducing both nonspecific and specific antitumor effects. As an adjuvant therapy, HT not only directly destroys tumor cells but also modulates the TME. It strongly induces HSPs, causes DNA damage, disrupts DNA repair pathways, triggers apoptotic cascades, and produces tumor neoantigens, which ultimately activates numerous immune cells and promotes infiltration of T-cells, B-cells, and NK cells into tumors. The innate immune response is initiated through the recognition of pathogen-associated molecular patterns (PAMPs) to TLRs, the prototypical pattern recognition receptors. Enhanced temperatures stimulate a more robust respiratory burst and promote infiltration of neutrophil granulocytes into tumors. HT also improves the phagocytic and bactericidal capabilities of macrophages. Following antigen uptake, DCs migrate to lymph nodes to present processed immunogenic peptides to naive T-lymphocytes. Systemic HT further enhances adaptive immunity to tumor antigens by promoting the maturation of DCs and upregulation of surface markers such as CD80, CCR7, and CD86. This leads to improved migration, antigen presentation, activation of antigen-specific T-cell responses, and cytokine-mediated stimulation of additional immune cells [2,4,6,13,79,90].

Cytotoxic T-cells eliminate tumor cells directly, either through secreted cytolytic factors or by inducing apoptosis via the Fas-FasL pathway. However, cytotoxic T-cell responses are often suppressed in tumors (lung, breast, pancreatic, cervical, prostate, etc.) due to the downregulation of MHC I molecules, which represents a strategy for evading the immune response. NK cells act as a second line of defense against tumor cells by recognizing cells with low MHC I expression. NK cells express both inhibitory and activating receptors, and the integration of these signals determines their overall immune response [13]. In healthy tissues, MHC I molecules maintain NK cells in a quiescent state by binding to their inhibitory receptors. In tumors, loss of inhibition, together with the presence of tumor-associated activating ligands (e.g., MICA, MICB, and ULBP), triggers a cytotoxic response against tumors with low MHC expression. In addition, HT modifies the expression of HSPs, which play vital roles in cancer therapy, including enhancing immune responses by improving the efficacy of immune checkpoint inhibitors [91].

Furthermore, numerous preclinical and clinical data indicate that HT alleviates the toxicity of combined chemotherapy and RT through immunomodulation and by increasing cytokines that recruit neutrophils, cytotoxic T-cells and dendritic cells, thereby reducing neutropenia [90] and stimulating the overall immune response [79,90,92]. Peripheral neutrophil counts, cytokine levels (including G-CSF and CXCL1/KC), and colony-forming unit (CFU) counts of bone marrow progenitor cells were significantly higher after HIPEC compared with conventional intraperitoneal (IP) chemotherapy. These findings suggest that HIPEC improves neutrophil and bone marrow recovery relative to standard IP chemotherapy [92,93,94]. In addition, HT demonstrates gastroprotective properties by preserving the integrity of the intestinal barrier and reducing gastrointestinal side effects, such as nausea, vomiting and diarrhea. This protective effect is mediated through the activation of autophagy and its regulatory mechanisms, which are important for maintaining intestinal homeostasis and facilitating repair. Furthermore, autophagy supports intestinal barrier function by regulating tight junctions, protecting epithelial cells from death, and maintains the intestinal epithelial barrier by supporting the function of specialized secretory cells like goblet and Paneth cells. It also plays a critical role in intestinal stem cells, influencing their metabolism as well as proliferative and regenerative capacity.

## 3. Hyperthermic Oncology

Extensive evidence shows that HT has a synergistic effect in cancer therapy when combined with RT, chemotherapy, or immunotherapy. Its therapeutic efficacy and low toxicity are achieved through several key mechanisms: (i) increased blood flow within the TME; (ii) enhanced lymphocyte infiltration and oxygenation; (iii) increased penetration of therapeutic agents and modulation of drug uptake by tumor cells; (iv) inhibition of DNA repair enzymes and reduction in acquired resistance to therapy; (v) induction of apoptosis or necrosis in tumor cells through heat shock; and (vi) altered expression of surface markers and release of cellular debris that serve as antigens to initiate antitumor immune responses [2,6,13,50,51,79,90,91,92,93,94].

Today, the multiple choice of HT application options, the variety of heating methods, and the ability to combine it with different forms of therapy, make HT a valuable clinical approach for treating local solid tumors (e.g., colorectal, prostate, and bladder cancers), locally advanced cervical cancer, peritoneal cancer and advanced metastatic cancers such as melanoma and head and neck cancer. Furthermore, HT in combination with RT and/or chemotherapy has shown promising results in rare tumor types, including Merkel cell carcinoma, primary pleural synovial sarcoma, and plasmacytoid dendritic cell leukemia. HT may be administered alone or in combination with RT or chemotherapy, using several heating techniques such as radiofrequency, microwave, infrared radiation, laser, or high-intensity focused ultrasound (HIFU).

### 3.1. Hyperthermia in Combination with Chemotherapy

HT is most commonly used together with RT and/or chemotherapy, gene therapy, surgery, and immunotherapy. Its major advantages include synergistic interactions with chemotherapy and radiation, low toxicity to normal tissues, and reduces the likelihood of resistance in tumor cells. In addition to its direct antitumor effects, HT shows an indirect effect by enhancing antitumor immunity. The combined use of regional HT with systemic chemotherapy has significant therapeutic potential, since the local distribution of heat can improve the cytotoxic activity of antitumor drugs within the targeted region. Strong evidence indicates the complementary effects of HT and antitumor drugs. Tumor cells residing in poorly perfused areas, such as the tumor core, often display relative resistance to systemic chemotherapy due to limited drug exposure. HT helps in overcoming this limitation, as it is more effective in killing cells under hypoxic and acidic conditions. Moreover, temperatures in poorly vascularized tumor regions are usually higher due to the reduced cooling effect of circulating blood. An additional advantage is that regional HT at 40–43 °C increases local blood flow and vascular permeability, key factors that facilitate drug uptake [20]. In vitro and in vivo studies demonstrate that the combination of HT and chemotherapeutic agents enhances the cytotoxicity of drugs such as CP, cyclophosphamide, ifosfamide, and mitomycin [95]. Optimal synergy between heat and chemotherapeutic agents typically occurs at temperatures between 40.5 and 43 °C, and drug administration prior to heating is generally the most effective approach. Although the exact mechanisms through which HT enhances drug cytotoxicity remain completely understood, several contributing factors have been proposed. These include improved drug delivery due to increased perfusion, enhanced cellular uptake, better drug interactions with their cellular targets (e.g., enhanced alkylation or increased DNA damage), induction of apoptosis, stimulation of antitumor immune responses, increased blood flow and reoxygenation via vasodilation, chemosensitization, inhibition of DNA repair processes, and reduced interstitial fluid pressure. In addition, HT may suppress tumor-derived VEGF production, thereby inhibiting endothelial cell proliferation, vascular remodeling, and extracellular matrix organization.

### 3.2. The Influence of Hypertemia on Reversing Chemotherapeutic Resistance

One of the major limitations of chemotherapy in cancer treatment is the development of multidrug resistance (MDR). This phenomenon was initially attributed to the overexpression of a 170 kDa transmembrane protein known as P-glycoprotein (Pgp) [39]. Pgp acts as an ATP-dependent efflux pump that expels chemotherapeutic agents from the cell immediately after they enter. Over time, additional transporter proteins involved in drug resistance have been identified, including multidrug resistance protein 1 (MRP1), lung resistance protein (LRP), and breast cancer resistance protein (BCRP). The key advantage of HT is its ability of reversing resistance to specific chemotherapeutics. HT enhances the cytotoxicity of drugs such as methotrexate, CP and mitomycin, even in cells that exhibit drug-resistant phenotypes [39,94,95]. Notably, cells displaying MDR through overexpression of Pgp or MRP1 do not exhibit cross-resistance to heat, which is a significant benefit from the clinical perspective [96]. As a result, HT can selectively target and destroy subpopulations of drug-resistant cells that survive chemotherapy due to high levels of Pgp and MRP1.

### 3.3. Hyperthermia and Radiotheraphy

As described in our previous work (see review paper [39]), radiation induces a wide range of changes in tumor cells through both direct and indirect mechanisms. Key mechanisms include increased production of ROS (OH•, HOO•, O_2_^−^•, and H_2_O_2_); DNA damage (SSBs, DSBs, DNA cross-links, and DNA-protein cross-links); protein modifications and lipid damage; genomic instability and mutations; inflammation mediated by IL-1, IL-6, iNOS, TNF-α, and MMPs; damage to hematopetic blood cells and immunosuppression; altered gene expression; and induction of cell death and senescence. Conversely, antioxidant defenses, such as GSH, are reduced. When combined with RT, HT amplifies these effects by increasing ROS production, inhibiting DNA repair enzymes, and enhancing DNA damage, especially formation of DSBs. HT serves as a radiosensitizer by disrupting repair pathways, including non-homologous end joining (NHEJ), homologous recombination (HR), and the alternative NHEJ process. According to Berg et al. [97], NHEJ is the primary DNA repair mechanism throughout the cell cycle, while HR functions exclusively during the S/G2 phase and relies on the sister chromatid for error-free repair. However, when HT impairs HR, cells are forced to rely on NHEJ, which is more error-prone and leads to an increased frequency of chromosomal rearrangements. Importantly, BRCA1 and BRCA2 proteins, the specific components of the HR pathway, are suppressed during heat shock [98]. Their degradation further sensitizes tumor cells to inhibitors of poly (ADP-ribose) polymerase 1 (PARP-1). In addition, heat disrupts the activity of DNA-dependent protein kinase (DNA-PK), a key component of the NHEJ pathway [4,99,100].

The serine/threonine kinase ATM is a central regulator of the cellular response to ionizing radiation. Activated by DNA DSBs, ATM is recruited to damage sites by the MRE11/RAD50/NBS1 complex and subsequently phosphorylates numerous downstream targets, including key tumor suppressors like p53, BRCA1, and H2AX. Through these actions, ATM maintains cellular homeostasis by regulating cell cycle arrest, apoptosis, senescence and metabolic reprogramming in a highly cell type-dependent manner. Heat shock also activates ATM, resulting in the phosphorylation of CHK1 and CHK2, p53, histone γ-H2AX, and MDM2, and further contributing to p53 activation. ATM plays particularly important role in DSB repair, especially within the HR pathway. It activates HR-specific repair proteins, including BRCA1, BRCA2, and PALB2, and promotes DNA-end resection, i.e., the generation of single-stranded DNA around the break site, which is a critical step for HR initiation.

The tumor suppressor p53 protein plays a crucial role in stopping the cell cycle in response to unrepaired DNA damage. In cells with wild-type p53, DSBs typically trigger a G1/S cell cycle arrest through the upregulation of its downstream effector p21. In contrast, cells with mutated p53 are unable to initiate this checkpoint and display G2/M arrest upon heat shock [101]. Taken together, these effects explain why HT significantly enhances the therapeutic efficacy of RT.

### 3.4. Inhibition of DNA Repair by Hyperthermal Treatment

Radiation and chemotherapy induce various types of DNA damage that can produce sublethal, potentially lethal, and fatal injury [39]. Most of the damage (first two) that occurs can be repaired and allow tumor cells to survive. However, efficient DNA repair mechanisms protect both healthy and cancer cells against the effects of treatment and contribute to the development of drug resistance. Inhibition of DNA repair mechanisms by hyperthermia results in irreversible DNA damage in tumor cells. Hyperthermia leads to the denaturation of cell proteins, including a variety of DNA repair enzymes, leading to increased sensitivity to radiation or chemotherapy (radio- and chemosensitization) [85,97,99,102,103,104]. The sensitivity of tumor cells to hyperthermia, chemotherapy, or radiation depends on the phase of the cell cycle, which may indicate a diversity of molecular mechanisms that trigger cell death and the involvement of different checkpoint mechanisms. In general, radiation is sensitive to the G2/M phase, while hyperthermia is sensitive to S-phase cells and during the M phase. The highest heat sensitivity can be observed during the M phase, with damage to the cellular mitotic apparatus leading to inefficient cell division and polyploidy. Therefore, this combination can significantly increase damage to all phases of tumor cells. The mammalian checkpoints started in response to DNA damage are managed by the two master kinases, ATM and ATR. ATM and ATR, as well as DNA-PK kinase, phosphorylate histone H2AX and other repair factors in chromatin domains, leading to the accumulation of multiple DNA repair-related proteins on damaged chromatin, propagation of damage signals, and activation of appropriate cell cycle checkpoints through mechanisms dependent on Chk1, Chk2, p53, CDC25a, WEE1, and other factors [103,104].

A summary of advantages and disadvantages of HT in combination with chemotherapy and RT is presented in Table 5.

## 4. Hyperthermia in Combination with Natural Compounds

Chemotherapy’s ability to disrupt cell integrity has been widely exploited in tumor treatment, leading to the development of a wide range of chemotherapeutic drugs with different mechanisms of action. Standard cancer therapies typically involve the usage of multiple chemotherapeutic agents to prevent cross-resistance in tumor cells. Although this strategy can increase treatment effectiveness, it may also lead to more severe side effects and poorer treatment outcomes [39].

In recent years, combined chemotherapy approaches have become increasingly common in the treatment of malignant diseases. These methods aim to enhance the antitumor immune response while minimizing toxicity to healthy cells. At the same time, there is a growing need to identify substances with protective effects, known as cytoprotectors, which are non-toxic to healthy cells and do not diminish the therapeutic efficacy of chemotherapy. These cytoprotectors help reduce the harmful effects of chemotherapy on normal tissues.

The use of plants and herbal preparations in the treatment and prevention of various diseases has been recognized in both traditional and classical medicine for centuries. Consequently, many plants have been investigated as potential protectants against the harmful effects of chemotherapy. Flavonoids, which are abundant in plants, plant-based diets and bee products, display potent antioxidant properties and represent natural, readily available, and generally non-toxic cytoprotectors. Numerous studies have examined their chemopreventive, immunomodulatory, and antitumor effects in animal models, particularly in tumor-bearing mice with weakened immune systems (see review paper [39,83,105]).

A newer therapeutic approach, combining chemotherapy, immunomodulation with natural products, and HT, has the potential to enhance tumor cell destruction while reducing toxicity to healthy tissues. This combined strategy may lead to improved treatment outcomes and better survival rates in tumor-bearing animals or humans.

### 4.1. The Use of Natural Compounds in Combination with Hyperthermia and Other Forms of Tumor Therapy

Epidemiological data indicate that diet strongly influences overall health and the development of chronic diseases. Natural products of plant origin with antitumor activity, such as propolis and its polyphenolic components, have long been used as medicinal preparations in the prevention and treatment of various conditions, including inflammatory diseases, cardiovascular disorders, diabetes, osteoporosis and tumors.

Propolis is a bee product that has been used in folk medicine since ancient times for the treatment of wounds and burns, sore throats, gastric ulcers, and other diseases. It is a complex mixture of resinous plant substances collected by bees from buds and tree bark, combined with beeswax and salivary gland secretions. Due to its antimicrobial, antiviral, antifungal, anesthetic, antiprotozoal, anti-inflammatory, antioxidant, antitumor, and immunostimulatory properties, propolis serves as protective agent not only for bees, but also for animals and humans. Its wide spectrum of biological activities has increased scientific and medicinal interests in both its chemical composition and diverse pharmacological effects. For this reason, propolis has become the subject of numerous chemical and pharmacological studies, including investigations into its potential contribution in tumor therapy, and the possibility of its use with chemotherapy, RT and HT with the aim of overcoming acquired resistance and reducing the toxic side effects of conventional cancer treatments. The direct antitumor effect of propolis and its flavonoid components is based on the ability to prevent the metabolic activation of carcinogens, inhibit proliferation and induce the apoptosis of tumor cells. Its indirect effects arise from powerful antioxidant and immunomodulatory activities. Importantly, differences between normal and tumor cells, particularly in baseline ROS levels, play a key role in their differential responses to various forms of therapy. The antitumor effects of propolis are described in detail in [83], while its potential to overcome resistance to chemotherapy and RT is discussed in [39].

In our previous paper [5], we listed numerous mechanisms through which propolis and its flavonoids exert chemopreventive effects and inhibit tumor growth. The anticancer activity of propolis and its components relies on various mechanisms, including cell cycle arrest, suppression of cancer cell proliferation, reduction in the CSC population, induction of apoptosis, modulation of oncogenic signaling pathways, inhibition of MMPs, prevention of metastasis, antiangiogenic activity, and anti-inflammatory effects. These direct antitumor effects are accompanied by the modulation of the tumor microenvironment, macrophage polarization, epigenetic regulation, antiviral and bactericidal activities, regulation of glucose metabolism, and modulation of gut microbiota.

### 4.2. Natural Compounds Increase the Chemosensitivity and Immunomodulation

Natural compounds, including propolis and its flavonoids, have demonstrated significant pharmacological potential as complementary or alternative therapies for the prevention and treatment of various cancers. These compounds may help overcome drug resistance or act as chemosensitizers in adjunct cancer therapy. Studies, including our own, have shown that propolis exhibits strong cytostatic, anti-carcinogenic, and antitumor effects in both in in vitro and in vivo tumor models [39,83]. The therapeutic activities of propolis are believed to dependent largely on its flavonoid content. Flavonoids have been reported to stimulate the immune system, scavenge ROS, and regulate numerous tumor-related processes, such as oxidative stress, cell proliferation, cell cycle progression, apoptosis, angiogenesis, and metastasis, by modulating various signaling pathways. Growing evidence suggests that flavonoids can target specific signaling pathways, including p53, EGFR, VEGF, apoptosis, phosphatidyl inositol-3-kinase (PI3K)/Akt/mammalian target of rapamycin (mTOR), extracellular signal-regulated kinase 1/2 (ERK1/2) (a subfamily of MAPKs), signal transducer and activator of transcription (STAT), nuclear factor kappa B (NF-κB), and Wnt/β-catenin pathways [1,2,3,4,5,6,7,8,9].

Flavonoids also exert antitumor effects by inhibiting pro-oxidative enzymes (e.g., xanthine oxidase, cyclooxygenase, and lipoxygenase), reducing polyamine synthesis through the inhibition of ornithine decarboxylase, and modulating protein kinases that regulate cell proliferation, differentiation and transformation (e.g., protein tyrosine kinase, protein kinase C, cAMP-dependent protein kinase, cyclin-dependent kinases, and MAPKs). In addition, they inhibit enzymes involved in DNA synthesis (including type I and II topoisomerases), and function as catalytic inhibitors, poisons, or DNA intercalators [83,106]. Furthermore, flavonoids can prevent tumor progression and malignant transformation by inducing apoptosis through the mitochondrial pathway, involving AIF, cytochrome C, and caspases. They modulate key regulatory proteins, increasing the expression of proapoptotic proteins Bax and p21 while reducing antiapoptotic Bcl-2 and p53 expression [39,83,107,108,109,110,111,112]. In addition to NF-ĸB, flavonoids regulate other transcription factors, including activator protein-1 (AP-1), which plays a role in cell proliferation and survival. They also modulate the expression of HSPs and inhibit VEGF [13,39,83].

Propolis and its flavonoids can reverse MDR and, in combination with cytostatics, exert additive and/or synergistic effects by increasing the chemosensitivity of cancer cells while reducing the cytotoxic effects on healthy cells. They also modulate the immune response within the TME through the regulation of cytokine/chemokine secretion, modulation of immune checkpoints and MHC I, regulation of NF-κB, AKT, and mTOR signaling pathways, as well as through the activation of NK cell receptors. Furthermore, their effect on the process of migration and invasion has been demonstrated through the modification of the EMT program and regulation of PI3K/AKT, Smad, NF-κB, JNK and ERK expression in tumor tissue. Flavonoids and propolis have also shown strong synergistic effect with chemotherapeutics by increasing their inhibitory impact on cancer cell proliferation, induction of apoptosis, cytochrome c release and modulation of Bcl-2 and Bax protein expression. In addition, they can reduce hypoxia-induced expression of HIF-1 and VEGF, which represent important targets in effective chemopreventive and antitumor strategies. Reduced levels of HIF-1 decrease the expression of the GLUT-1 transporter, consequently reducing glucose uptake into tumor cells, lactate production, Akt and mTOR signaling, and cell viability in a dose- and time-dependent manner. Flavonoids can also inhibit histone deacetylase (HDAC) classes I, II and IV, contributing to cell cycle arrest, inhibition of angiogenesis, and induction of cell death. Moreover, by promoting p53 acetylation, flavonoids increase the expression of p53 target genes involved in growth arrest and apoptosis [13,39,83].

When combining propolis or flavonoids with various forms of cancer therapy (such as chemotherapy, RT, and HT), the above-described processes can significantly contribute to overcoming chemo- and radioresistance. HT can additionally help overcome the resistance of tumor cells to chemotherapy and RT, but also reduce the doses of applied therapies, thus protecting normal healthy cells. Hyperthermia can increase the cytotoxicity of cancer cells and stimulate the immune response through the activation of immune cells, act on immune checkpoints, modulate the local proinflammatory response, induce cell death and cause changes in the microenvironment mainly through the increase in specific HSPs [113,114,115,116]. Additionally, HT may be useful in overcoming resistance to chemotherapy drugs, such as CP, doxorubicin, 5-FU, mitomycin, docetaxel, by increasing drug uptake and altering tumor microcirculation, blood flow, cell membrane permeability, and cellular metabolism, thus confirming its potent chemosensitizing effects [117,118,119].

In this paper, we will explore the mechanisms by which propolis and flavonoids enhance sensitization to chemotherapy and RT with the use of hyperthermia treatment. We will highlight the positive and negative effects on healthy and tumor cells using multimodal therapy, immunotherapy with propolis and flavonoids, hyperthermia, and chemotherapy and RT.

The integration of HT with propolis-based interventions opens the possibility of achieving a more selective therapeutic response against cancer. In the following sections, we will summarize the current findings on the molecular and cellular mechanisms involved, discuss their relevance in overcoming therapy resistance, and outline the potential benefits and limitations of this multimodal approach.

### 4.3. Natural Compaunds in Combination with Hyperthermic Intraperitoneal Chemotherapy

Hyperthermic intraperitoneal chemotherapy (HIPEC) is a form of cancer therapy used for tumors spread within the abdominal cavity (peritoneum). After surgical removal of the visible tumor tissue, the goal of HIPEC is to eliminate remaining cancer cells and reduce the risk of recurrence. As explained previously, HT potentiates the cytotoxicity of several antitumor agents, including bleomycin [120], cyclophosphamide [121], mitomycin C [117,118,119,122], CP [9,10,13,95,117,118,119], doxorubicin [119,123,124], 1,3-bis(2-chloroethyl)-1-nitrosourea [125,126], and gemcitabine [127]. When applied prior to conventional therapy, hyperthermia can increase blood flow, improve oxygenation and enhance the therapy effects by stimulating production of oxygen radicals [13,95].

Hyperthermia is a promising approach to cancer therapy because it not only kills cancer cells directly, but it also activates anticancer immunity as an indirect effect [2,4,6,13,90,95,118]. In fact, the mechanism(s) by which hyperthermia kills cells remains controversial [9,128], as does the means by which elevated temperatures increase the cytotoxicity of anticancer drugs. Four general possibilities which have been proposed are as follows: (i) increased levels of drugs within cells [13,54,55,95,117,118,119]; (ii) increased efficiency of lesion formation [9,10,13,95,129,130]; (iii) occurrence of different lesions at hyperthermic temperatures; and (iv) heat-induced inhibition of DNA repair [85,95,97,99,103,104,130]. Moreover, the same findings indicate that heat shock proteins induced in tumor cells under hyperthermic stress are able to elicit specific T- and NK cell immune response [2,4,6,13,79,90,95].

Oršolić et al. [9,10,95] designed several studies to examine whether combining immunotherapy with HIPEC could improve survival in mice, and which therapeutic approach would be the most effective in preventing peritoneal carcinomatosis (PC), an advanced form of cancer. The chemopreventive, immunomodulatory, cytotoxic and genotoxic effects of aqueous propolis solution (WSDP), alcoholic propolis solution (AOP), quercetin (QU) and naringenin were evaluated in both preventive and therapeutic protocols together with CP in a Swiss albino mice-bearing Ehrlich ascites tumor (EAT) in physiological (37 °C) and hyperthermic (43 °C) intraperitoneal conditions. WSDP combined with HIPEC increased mouse survival by an additional 160.3% compared with HIPEC alone. WSDP reduced the toxic and genotoxic effects of CP in normal cells without diminishing CP cytotoxicity in EAT cells. In addition, WSDP combined with HIPEC enhanced the cytotoxic activity of macrophages toward tumor cells. These findings demonstrate that the WSDP increases macrophage activity, enhances the sensitivity of tumor cells to HIPEC, and reduces CP toxicity in healthy tissues. The proposed mechanisms underlying these effects include: (i) increased intracellular drug levels, (ii) improved efficiency of lesion formation, (iii) the appearance of distinct lesions at hyperthermic temperatures, and (iv) heat-induced inhibition of DNA repair. In addition, HSPs induced in tumor cells under hyperthermic stress may further contribute to antitumor effects by stimulating T-cell and NK cell responses.

Elevated temperatures (40–44 °C) selectively kill tumor cells due to fundamental physiological differences between malignant and healthy tissues. Tumor vasculature is highly disorganized and inefficient, resulting in areas of hypoxia and low pH that are largely absent in normal tissues. These environmental features render tumor cells more sensitive to hyperthermic stress. HT amplifies the cytotoxic effect of CP by promoting its cellular uptake and accumulation, increasing the formation of adducts with intracellular targets, facilitating the appearance of various DNA lesions, and inhibiting DNA repair mechanisms. HT also enhances CP solubility and its interactions with DNA, predominantly resulting in intrastrand cross-link adducts that activate apoptotic pathways. Consequently, HT reduces the number of tumor cells, improving the survival of tumor-bearing mice. Furthermore, exposure to elevated temperatures induces structural and functional alterations in cells. These include increased passive and active transport across damaged membranes, inhibition of protein and enzyme synthesis, disruption of lysosomal integrity, and impairment of the mitotic spindle. HT additionally affects the activity of key enzymes, such as DNA and RNA polymerases, and suppresses DNA synthesis and repair, further potentiating the antitumor effects of CP. WSDP also exerts immunomodulatory effects that may contribute to tumor suppression. It enhances the activity of several immune cells, especially macrophages, dendritic cells, T-cells and NK cells. The reduction in tumor cells appears to result from both the direct cytotoxic effects of propolis and CP and the immunostimulatory properties of propolis. A macrophage spreading assay revealed that WSDP affects the functional state of macrophages, which have the central role in tumor cell elimination. Moreover, cytokines secreted by activated macrophages, especially under hyperthermic conditions, such as IL-1, IL-6, IL-12, and TNF-α, together with NO, can exert direct cytotoxic effects on tumor cells. Additional cytokines activate other components of the immune system, including cytotoxic T-cells, B-cells, and NK cells [9,10,95]. These cytokines may also promote the production of C-reactive protein and complement component C3, which can act as opsonins and enhance recognition of tumor cells. Taken together, these processes may slow tumor growth and facilitate the elimination of tumor cells [95].

### 4.4. Natural Compaunds Increase the Clastogenic Activity of Alkylating Agents Under Hyperthermal Conditions

It is well known that HT enhances the clastogenic potential of alkylating agents. Asanami and Shimono reported a synergism between HT and alkylating agents, such as mitomycin C (0.1–0.5 mg/kg) and cyclophosphamide (1.25–10 mg/kg), while colchicine, spindle poison, and the antimetabolite 5-fluorouracil (2.5–50 mg/kg) produced an additive effect [131]. This synergism appears to result from the induction of DNA lesions and the reduced ability of cells to repair damage. HT also disrupts the mitotic apparatus and induces the formation of micronuclei (MNs) [132].

The clastogenic effect of CP is based on its covalent binding at the N-7 position of purine residues, leading to the formation of 1, 2 or 1, 3-intrastrand cross-links, with a smaller proportion of interstrand cross-links. Interestingly, propolis and its major flavonoids, QU and naringenin, when administered with CP, inhibited tumor growth in both normothermic and hyperthermic conditions in mice. However, they decreased the number of MNs in the peripheral blood reticulocytes under normothermic condition, but enhanced the clastogenicity of CP in hyperthermal conditions [9,10]. This paradoxical increase can be explained by HT-induced alteration of cellular physiology. Elevated temperatures increase both passive and active membrane transport and enhance binding to DNA. Furthermore, HT increases production of ROS, intensifying oxidative stress. High doses of CP are known to induce nephrotoxicity, hepatotoxicity, neurotoxicity, myelosuppression and genotoxicity. These effects are closely associated with increased generation of ROS, such as hydroxyl radicals and superoxide anions. Under physiological conditions, intake of propolis and flavonoids has a protective effect on liver, kidney and blood cells, likely due to their numerous antioxidant abilities. These include scavenging of free radicals, metal chelation, inhibition of prooxidant enzymes, detoxification and upregulation of antioxidant enzymes, anti-inflammatory effects, and reduction in capillary and venular permeability [9,10].

### 4.5. The Pro-Oxidative Effect of Natural Compaunds Increases Hyperthermia-Induced DNA Damage and Promotes Tumor Cell Apoptosis

Although flavonoids are recognized for their antioxidant properties, they can also exert pronounced pro-oxidant effects that contribute to apoptosis and genotoxicity of tumor cells [83,95,133]. Flavonoids show pro-oxidative effects by reducing ferric iron (Fe^3+^) to ferrous iron (Fe^2+^), which subsequently catalyses the conversion of H_2_O_2_ into highly reactive hydroxyl radicals via Fenton chemistry, thereby promoting oxidative damage. When directed toward cancer cells, this pro-oxidant activity can serve as an anticancer mechanism, as it may trigger cell death. Excessive ROS accumulation by flavonoids (epigallocatechin gallate (EGCG), hesperetin, naringenin, QU, resveratrol, luteolin and apigenin) can activate apoptotic, autophagic, and necroptotic pathways, leading to reduction in tumor size [9,10,13,95,133]. Conversely, due to their antioxidant properties and ROS scavenging abilities, flavonoids may prevent cancer initiation in normal cells. Besides generating ROS and initiating apoptosis, flavonoids exert additional antitumor effects, such as the inhibition of glycolysis, interference with enzyme synthesis, binding to estrogen type II sites, and modulation of molecular targets along signaling pathways. Flavonoids such as QU and myricetin, particularly in the presence of Fe^2+^ and increased ROS levels induced by CP, increase CP-mediated cytotoxicity in cancer cells. The auto-oxidative activity of QU may arise from its high intracellular concentration and its inhibitory effect on mitochondrial respiration, which leads to increased production of superoxide anions and H_2_O_2_. In turn, H_2_O_2_ contributes to DNA strand breakage by generating hydroxyl radicals in close proximity to DNA molecules via the Fenton reaction. It should also be noted that flavonoids may inhibit DNA repair processes by inhibiting poly(ADP-ribose) polymerase (PARP), a key enzyme participating in DNA repair following alkylation-induced damage in both normal and cancer cells [134].

Lu et al. [135] introduced a novel strategy termed thermal cycling–hyperthermia (TC-HT), designed to maximize synergy with anticancer compounds while minimizing side effects. Using this approach, they demonstrated that TC-HT acts synergistically with polyphenols EGCG and chlorogenic acid (CGA) in PANC-1 pancreatic cancer cells. Co-administration with TC-HT and either EGCG or CGA significantly inhibited cell proliferation, triggered cell cycle arrest at the G2/M phase, and activated the mitochondrial apoptotic cascade characterized by an imbalance between Bcl-2 and Bax, activation of caspase-9 and -3, and PARP cleavage. Neither 10 cycles of TC-HT (43.5–36 °C) nor treatment with polyphenols alone produced a substantial change in cell viability. However, the combination of 10 cycles of the TC-HT with CGA (200 μM) or EGCG (20 μM) resulted in a pronounced decrease in cell viability. Similar results were obtained in colony formation assays. These findings indicate that TC-HT may synergize with natural polyphenolic compounds to produce robust anticancer effects while simultaneously minimizing thermal damage resulting from HT [135].

A substantial reduction in chemotherapy or RT doses, when combined with TC-HT, can enhance the anticancer efficacy of the herbal compound echinacoside (Ech) against PANC-1 cells [136]. Echinacoside, a natural phenylethanoid glycoside originally extracted from *Echinacea angustifolia*, exhibited multiple anticancer mechanisms when combined with TC-HT, acting on several key proteins that regulate apoptosis, including Bcl-2 and MAPKs. Ech also inhibited the catalytic activity of MutT homolog 1 (MTH1), an enzyme that detoxifies oxidized dNTPs and is usually highly expressed in various cancer cells. TC-HT amplified Ech-induced apoptosis, allowing for a reduction in the required dose. The synergistic effect of this combination was visible in suppressed expression of MTH1, resulting in the accumulation of 8-hydroxy-2′-deoxyguanosine triphosphate (8-oxo-dGTP), increased DNA damage and activation of mitochondrial apoptotic pathway. The combined treatment also promoted PARP cleavage, thereby impairing DNA repair mechanism in PANC-1 cells. The study also showed that treatment with 20 μM Ech in combination with TC-HT can achieve the same level of inhibition as 100 μM Ech alone. This dose reduction not only enhanced the antitumor efficacy but also reduced the harmful effects on healthy cells [136].

Kuo et al. [137] applied low-intensity ultrasound (US) as an additional adjuvant to TC-HT to enhance the anticancer effect of propolis. This triple treatment markedly reduced the viability of PANC-1 cells, achieving an efficacy comparable to that of standard chemotherapeutics but without inducing damage to normal cells. The incorporation of low-intensity US in combination with TC-HT and propolis altered the phosphorylation status of MAPK family members, elevated intracellular ROS levels, and activated mitochondrial apoptotic pathways in PANC-1 cells. Among all tested approaches, this triple treatment induced the highest rate of apoptosis, indicating that excessive intracellular ROS accumulation is the main driver of cell death under these conditions. Mitochondrial dysfunction was induced via MAPK modulation, triggering apoptosis through enhanced PARP cleavage. The application of CP alone (1 µM) had minimal effect on PARP cleavage in PANC-1 cells, whereas the combination of CP with TC-HT markedly increased PARP cleavage (3.20-fold). The addition of US further amplified this effect by 5.82-fold. These findings indicate that triple treatment significantly increased caspase-induced PARP cleavage, surpassing the efficacy of combination of propolis and TC-HT alone. Taken together, it seems that combining propolis, TC-HT and low-intensity US represents a promising anticancer approach to enhance the anticancer efficacy of the heat-sensitive chemotherapy drug CP while enabling dose reduction [137].

Oršolić et al. [117] demonstrated that combined treatment with the plant extract *Caucalis platycarpos* (CPL; 200 mg/kg), rich in polyphenolic/flavonoid compounds, and cytostatic agents (CP 10 mg/kg, doxorubicin 20 mg/kg, and mitomycin 5 mg/kg) significantly inhibited the development of peritoneal carcinomatosis and increased the survival of mice. These results suggest the synergistic effect of hyperthermia, chemotherapy, and immunotherapy. CPL significantly increases the antitumor activity of the hyperthermic chemotherapy and the survival rate of mice with peritoneal carcinomatosis. The stimulatory effect of CPL on the immune response, particularly T- and NK cell activity, protects mice from the development of peritoneal carcinomatosis and reduces the side effects of chemotherapy, increasing the lifespan of mice [117]. Furthermore, it has been shown that preventive treatment with QU increased survival in mice treated with HIPEC (CP 5 mg/kg) by an additional 159.46% compared with HIPEC alone (186.54%) [9].

In another study [95], the differential sensitivity of T24 and UMUC human bladder cancer cells was observed following short-term exposure to QU (2 h) and CP (1 h). The effects of both compounds were evaluated at low and high micromolar concentrations (1 and 50 µM, respectively) under physiological and hyperthermic conditions. QU acted in either an additive or synergistic manner in combination with CP across both temperature conditions. As determined by a 3-(4,5-dimethylthiazol2-yl)-2,5-diphenyltetrazolium bromide (MTT) assay, short-term exposure to QU and CP reduced cell viability. A clonal assay also indicated that the combination of QU and CP was lethal to bladder cancer cells under both normothermic and hyperthermic conditions. This short-term exposure protocol to QU and CP was designed to mimic clinical intravesical therapy, suggesting potential applicability for bladder cancer treatment under physiological and hyperthermic conditions. Translating these findings in vivo implies that high local concentrations of CP can be achieved in the bladder without a corresponding increase in CP in serum, thereby reducing systemic toxicity. HT may further enhance chemotherapeutic efficacy by increasing CP uptake, altering intracellular distribution and metabolism, and inhibiting DNA repair in bladder cancer cells. All of these effects improve the overall antitumor activity of chemotherapeutic agents [95]. Furthermore, it has been demonstrated that QU acts as a hyperthermic sensitizer in HeLa cells, melanoma cells, and lymphoid leukemia and ovarian cell lines by inducing intracellular lactate accumulation and acidification [95,138,139]. QU also inhibits tumor growth and amplifies the effects of hypothermia in PC-3 and DU-145 prostate tumor cells [138,139].

In vivo, this multimodal approach exerted synergistic antitumor effects under both temperature conditions. Increased efficacy in vivo was demonstrated through the reduced number of cancer cells in the abdominal cavity, prolonged survival of treated mice, increased genotoxicity (including sister chromatid exchanges, chromosome aberrations, and MNs formation), as well as increased apoptosis, necrosis and immune responses.

Unlike in vitro studies, in vivo models offer additional advantages, as they incorporate various components of the immune system, increasing immune surveillance and providing protection against tumor growth. These models also include additional antitumor mechanisms, including antiestrogenic, antiangiogenic and proapoptotic/necrotic mechanisms, selective destruction of tumor cells in hypoxic and low pH environments, and the potential to reverse resistance to certain chemotherapeutic drugs [10,133,138,139,140,141,142,143,144,145,146,147,148,149,150].

Based on our data and the literature, the anticancer activity of CP is achieved through several mechanisms: (i) QU increases immune responses and direct DNA damage induced by apoptotic processing; (ii) QU enhances the accumulation and penetration of CP at the tumor site, thus remodeling the microenvironment; (iii) QU acts as a hyperthermic sensitizer, promoting intracellular lactate accumulation and acidification, which increases lysosomal activity under low pH levels and intensifies heat-induced cell membrane damage; (iv) QU triggers apoptosis and cell cycle, sensitizing tumor cells to CP; (v) QU stimulates ROS production, inducing mitochondrial dysfunction and downregulating antiapoptotic factors, such as Bcl-2 and Bcl-XL, while upregulating proapoptotic proteins, like Bax and Bid; (vi) QU inhibits MRPs, reducing CP efflux from cancer cells; (vii) QU suppresses HSP27, HSP70, HSP72 and HSP90, stimulates apoptosis via ER stress, and inhibits STAT3 phosphorylation; (viii) QU prevents formation of new blood vessels; (ix) QU acts synergistically with CP via different mechanisms (interferes with topoisomerases I and II, interacts with estrogen receptor type II binding sites, and modulates various signaling pathways involved in cell proliferation, complementing CP effects on the formation of DNA adducts and ROS; (x) QU affects miRNAs, NF-κB, MAPsK, AMPK, and HIF pathways, enhancing the cytotoxic effects of CP; (xi) QU inhibits protein kinase C δ (PKCδ), enhancing the cytotoxic effects of CP and hyperthermia, while protecting kidney cells [9,10,133,138,142]. In addition, QU contributes to the protection of healthy tissues against CP-mediated toxicity. In the kidney and liver, QU reduces vascular permeability and plasma volume loss after thermal injury. It also protects RBCs from deformation, prevents platelet aggregation, and stabilizes lysosomal membranes [9,95,138,143]. Furthermore, QU provides free radical scavenger activity, increasing the activity of SOD, catalase and GSH in healthy cells [9,95,138].

### 4.6. Flavonoids as HDAC Inhibitors and Modulators of HSPs in Hyperthermal Chemotherapy

As reported by Masoud et al. [151] and Kim et al. [152], hypoxia enhances HDAC activity, which contributes to angiogenesis through suppression of hypoxia-responsive tumor suppressor genes. Various stressors, including chemical agents, elevated temperatures, free radicals, and mechanical stimuli, can trigger HSP response, and HDAC inhibitors can regulate functional activity of these proteins. Thus, fisetin, a dietary flavonoid, induces apoptosis in cancer cells by suppressing heat shock factor 1 (HSF1), the transcriptional regulator of HSP expression, by blocking its binding to the hsp70 promoter [153]. When the HCT-116 cells were exposed to heat shock in the presence of fisetin, the induction of HSF1 target proteins, such as HSP70, HSP27 and BAG3 (Bcl-2-associated athanogene domain 3), were inhibited. The downregulation of HSP70/BAG3 by fisetin significantly reduced the amounts of Bcl-2, Bcl-xL and Mcl-1 proteins, subsequently inducing apoptotic cell death. Intraperitoneal treatment of nude mice with fisetin at 30 mg/kg resulted in a 35.7% (*p* < 0.001) inhibition of tumor growth [153]. HDAC inhibitors markedly suppress HIF-1α expression and are currently evaluated in clinical trials, partly due to their potent antiangiogenic properties [154].

In the context of improving cancer treatment options, there is growing interest in the scientific community towards bioactive natural compounds with HDAC-inhibitory potential. Resveratrol, a naturally occurring polyphenol, inhibits all eleven human HDACs of classes I, II, and IV in a dose-dependent manner. HDAC inhibition is known to trigger various antitumor mechanisms, including cell cycle arrest, inhibition of angiogenesis, and induction of cell death [155]. Furthermore, resveratrol promotes p53 acetylation, thereby upregulating the expression of p53 target genes involved in cell death and growth inhibition [155,156,157]. Narita et al. [158] demonstrated that inhibition of histone deacetylase 3 (HDAC3) promotes heat shock-induced apoptosis under acidic conditions in human maxillary carcinoma cells, suggesting HDAC3 as a potential target for HT-based therapies. Accordingly, combined treatment including HT and HDAC inhibitors may represent a promising therapeutic approach.

In the study by Kučan et al. [13], a preventive treatment strategy using resveratrol, in combination with systemic HT and CP, was evaluated. The aim was to enhance CP-mediated antitumor effects through the modulation of HDAC activity and regulation of HSP70 and HSP90. These findings clearly demonstrated that HT increases CP toxicity, reduces body weight and improves treatment outcomes by inhibiting angiogenesis and tumor progression, resulting in a significant extension of animal lifespan. Importantly, the observed reduction in tumor mass was not exclusively related to the direct cytotoxic effects of CP, but was also associated with the inhibition of tumor growth and angiogenesis. This CP + HT treatment group had the smallest tumor volumes among all experimental groups, accompanied by decreased VEGF levels, the highest percentage of tumor growth inhibition, and a marked suppression in HDAC activity relative to controls. Similar results were observed in the Res + CP + HT group considering tumor growth inhibition, %ILS, VEGF levels and significantly suppressed HDAC activity. A particularly important finding of this study is that the combined treatment with CP, HT and resveratrol resulted in robust inhibition of HDAC activity, prevented the binding of HIF-1α to DNA, and altered expression of HSP70 and HSP90 [13]. These results indicate that suppression of HIF-1α signaling and inhibition of tumor angiogenesis are mediated, at least in part, by impaired nuclear translocation of HIF-1α. This interpretation is consistent with the study of Venturelli et al. [155], who suggested that HDAC inhibitors exert anti-HIF activity by destabilizing HIF-1α and suppressing the transactivation potential of its C-terminal activation (HIF-αCAD). Taken together, these findings support the concept that a multimodal approach combining resveratrol and CP with HT increases antitumor efficacy. This conclusion is in line with our previous observations and literature data [13,159,160,161,162,163].

Growing evidence indicates that plant-derived compounds, like sulforaphane, chrysin, pomiferin, QU, silibinin, luteolin, genistein, galangin, apigenin, EGCG, and curcumin, show HDAC inhibitory activity (see the reviews by [83,164]). Flavonoids, terpenoids, phenolic acids, and alkaloids significantly suppress HDAC activity and display promising epigenetic drug-like properties, supporting the concept that HDAC-targeting epigenetic drugs may be effective in the prevention and treatment of various human cancers. For example, apigenin has attracted much attention as an HDAC inhibitor, with prominent efficacy in prostate cancer models. Apigenin induces apoptosis by reducing antiapoptotic proteins and interfering with NF-κB signaling. Furthermore, it exhibits synergistic effects when used in combination therapy and is capable of triggering apoptosis even in prostate cancer models resistant to conventional therapeutic regimens [165,166].

The ability of tumor cells to gain self-sufficiency in growth signaling is closely associated with the activity of HSPs. Many proteins involved in the proliferation signaling pathway, such as the epidermal receptor growth factor (EGFR), human epidermal receptor growth factor 2 (HER2) and their downstream effectors such as RAS, RAF, ERK and AKT, are client proteins of HSP90. Thus, pharmacological targeting of HSP90 represents a potential strategy for the impairment of oncogenic signaling. Curcumin and its synthetic analog C0818 have been shown to inhibit both the PI3K/AKT and RAS/RAF/MEK/ERK signaling pathways in a proteasome-dependent manner, thereby inhibiting the proliferation of hepatocellular carcinoma (HCC) cell lines HepG2 and Sk-Hep1 [167]. This effect was accompanied with reduced levels of Cdc2, cyclin B1, and Cdc25c, together with increased levels of the cyclin-dependent kinase inhibitor p21, indicating that C0818 induces cell arrest in the G2/M phase. Similarly, demethoxycurcumin (DMC), a curcuminoid isolated from turmeric, reduced HER2 expression by disrupting the binding of HSP90 to HER2 and demonstrated pronounced antitumor effects in HER2-overexpressing RT4 bladder cancer cells.

QU is a well-known inhibitor of HSP70 synthesis. It suppresses the production of cytoprotective HSPs, sensitizing cancer cells to heat and chemotherapy, while simultaneously promoting apoptosis. QU in combination with tamoxifen exhibited strong synergistic effects with HT (42.5 °C) in reducing the clonogenic activity of M14 and MNT1 cells, and enhanced apoptotic cell death in M10, M14 and MNT1 human melanoma cells lines. This combinatorial treatment reduced HSP70 expression at both the protein and mRNA levels, supporting its potential usage in HT-based therapeutic strategies for recurrent and/or metastatic melanoma [168].

### 4.7. Targeting Cancer Stem Cells with Natural Compounds and Hyperthermia: A Multimodal Strategy to Overcome Resistance

CSCs are characterized by their self-renewal ability, enhanced EMT capability and drug resistance, largely due to the overexpression of ATP-binding cassette (ABC) transporters such as ABCB1, ABCG2, and ABCB5, as well as enhanced tumorsphere formation due to the aberrant activation of Wnt, Hedgehog, and Notch pathways. Therefore, the inhibition of ABC transporters by various natural compounds, including flavonoids, has emerged as a potential approach in the prevention of multidrug resistance and elimination of CSCs.

Propolis and flavonoid compounds have been shown to suppress acquired chemoresistance by interfering with the NF-κB-IL6 or NF-κB-ABC pathways. These effects are mediated through the downregulation of drug efflux pumps and antiapoptotic proteins (survivin and XIAP), the inhibition of the NF-κB signaling cascade, and the reduction in CSC progenitor formation. Additional mechanisms include the increased cellular uptake of chemotherapeutic agents, epigenetic mechanisms, the upregulation of proapoptotic factors (DIABLO and APAF1), and the regulation of multiple oncogenic signaling pathways and enzymes [39,83,139]. An important aspect of targeting CSCs is the modulation of the TME through macrophage polarization and Th1 activity. Propolis and related flavonoids influence immune responses by promoting macrophage polarization toward M1 phenotype and enhancing Th1-mediated cellular immunity. By reducing TAMs, propolis and its components may overcome EMT-mediated chemoresistance, disrupt the crosstalk between macrophages and CSCs, suppress stemness maintenance, and reverse acquired immunosuppression. These effects promote antitumor responses mediated by cytotoxic T-cells and improve therapeutic efficacy.

Propolis and flavonoids (QU, luteolin, apigenin, resveratrol, myricetin, wogonin, chrysin, hesperetin, daidzein, sylymarn and others) can enhance the efficacy of chemotherapy by overcoming CSC-mediated resistance and sensitizing tumors to treatment. HT complements these effects by directly killing cancer cells while simultaneously enhancing chemotherapeutic efficacy [83,139].

Interestingly, HT in combination with chemoimmunotherapy may reduce CSC-resistant cells [86,169]. CSCs are particularly resistant to conventional therapy and are the main drivers of disease recurrence.

Finally, the combined application of flavonoids, HT, and chemotherapy has the potential to improve anticancer efficacy through several complementary and synergistic mechanisms: (i) direct targeting and elimination of CSCs; (ii) increased chemosensitivity of tumor cells driven by flavonoid–HT interactions; (iii) suppression of key signaling cascades involved in inflammation, proliferation, angiogenesis, survival, and metastasis (e.g., PI3K/AKT/mTOR, JAK/STAT3, MAPK/ERK, and Wnt/β-catenin); (iv) modulation of the TME and immune responses; and (v) enhanced activation of regulated cell death pathways, including apoptosis, necrosis, autophagy, pyroptosis, and ferroptosis [83,170].

Moreover, HT may establish a favorable TME that facilitates an immune-mediated cascade, strengthening the therapeutic efficacy of combined CP and resveratrol treatment. By increasing local blood flow, HT increases the intratumoral accumulation of CP and remodels the TME by releasing danger-associated molecular patterns and tumor-derived antigens. These signals contribute to the reactivation of macrophage polarization toward the proinflammatory M1 phenotype and stimulate antitumor immune responses. Overall, treatments with CP + HT and Res + CP + HT have shown the most pronounced antitumor effects in vivo, although these outcomes appear to result from partially distinct synergistic/antagonistic mechanisms. Resveratrol contributed to subtle differences in animal survival, altered MMP-2 levels, and a marked reduction in arginase activity, thus affecting HIF-NO signaling. Specifically, intracellular NO levels and NO-mediated signaling play an important role in HIF-1 stabilization and activity, emphasizing the need to better understand the regulation of these pathways within tumor cells [13,76,77].

HIF-NO signaling is involved in a wide range of physiological and pathological processes, including development, angiogenesis, immune regulation, programmed cell death, survival, and aging [76,77]. As a powerful antioxidant, resveratrol may alter the balance between HIF and NO signals, thereby influencing angiogenesis, apoptosis, tumor progression and survival. Hypoxic conditions lead to the altered expression of more than 1000 mammalian genes, largely driven by increased HIF-1 activity. While HIF-1 enables cellular adaptation to hypoxia, its stability is negatively regulated by oxygen (O_2_). In addition, several other factors are impacted, including NO, modulate HIF-1 stability and transcriptional activity. NO generated from L-arginine and nitrite (NO_2_^–^) can nitrosylate or nitrate HIF-1 and associated regulatory proteins, allowing HIF-1 to avoid normoxic degradation. Conversely, HIF-1 can increase NO production through multiple mechanisms, including the upregulation of iNOS and cytochrome c oxidase subunit 4-2 (COX4-2). This mutual regulation highlights the extensive crosstalk between HIF-1 and NO signaling. Consequently, many cellular responses to NO are mediated by HIF-1, and vice versa. These processes include, but are not limited to, angiogenesis, apoptosis, aging, and metabolic reprogramming. O_2_ availability also shapes ROS/RNS signaling, as elevated NO production together with reduced ROS levels favors thiol nitrosylation, potentially modifying HIF-1α, PHD2 and SIRT1 [76,77].

Quantitative analysis of HIF-1α revealed higher levels in the Res + CP, CP + HT and Res + CP + HT groups, indicating increased cell death by apoptosis or necrosis, consistent with reduced tumor volume and increased animal survival.

### 4.8. Hyperthermia as an Adjuvant in Chemoimmunotherapy with Natural Compounds Modulates the Tumor Immune Microenvironment via Macrophage Activation and Inhibition of the PD1/PD1L Axis

A growing body of research indicates that HT, when applied as an adjuvant approach to cancer immunotherapy, is associated with an increased infiltration of monocytes and macrophages into tumor tissue and increased macrophage activity [9,10,51,171]. These changes correlate with tumor regression and improved therapeutic outcomes [9,10,51,171,172]. Our data demonstrated increased immunomodulatory activity of macrophages following treatment with HT in combination with propolis or its major flavonoids, such as QU, naringenin and resveratrol, highlighting the pivotal role of the immune system in tumor rejection [9,10,13,43,95].

Several molecular mechanisms of flavonoids may account for the enhanced immune reactivity induced by HT, including the induction of HSPs, enhanced activation of antigen-presenting cells, and alterations in lymphocyte trafficking. As mentioned, HSPs are involved in antigen processing and presentation and may act as endogenous “danger signals”. The immunogenic potential of HSP70 relies on its ability to bind tumor-derived peptide antigens [6,9,10,171,173]. The formation of the HSP70–peptide complex results in an antigen-specific CD8+ T-cell response. To recognize tumor cell antigens, CD8+ T-cells require the expression of MHC class I antigens, whose expression was reduced or completely abolished in as many as 40–90% of primary tumors and metastases. This downregulation helps tumors avoid the immune response. The lack of MHC-class I molecules makes tumor cells “invisible” to CD8+ T-cell-mediated toxicity. Conversely, the absence of MHC class I expression increases tumor susceptibility to NK cells. Activated NK cells expressing HSP70 receptors, such as the C-type lectin receptors CD94/NKG2C and NKG2D, can recognize membrane-bound HSP70 on tumor cells and induce cell death through the release of granzyme B, either alone or in combination with perforin [60]. In addition to their direct cytotoxic effects, chemotherapeutic agents such as CP, doxorubicin, mitomycin C, 5-fluorouracil and camptothecin exhibit indirect cytotoxic activity by “priming” tumor cells for immune-mediated elimination. This priming enhances sensitivity to death by NK cells or cytotoxic T-lymphocytes via death receptor-dependent pathways, such as Fas or the TNF-related apoptosis-inducing ligand (TRAIL) receptor pathways [39,174].

In addition, the maturation of DCs in the presence of platinum drugs reduces their PD-L1 and PD-L2 levels and increases the potential for T-cell activation [171].

Likewise, it has been shown that resveratrol may induce immunogenic cell death by promoting the surface expression of calreticulin (CRT) and high-mobility group box 1 (HMGB1) in ovarian cancer cells, resulting in the inhibition of tumor growth and volume. These effects were accompanied with the suppression of TGF-β and the concomitant increase in IL-12 and IFN-γ levels, indicating a shift towards proinflammatory immune response [43,157,175]. In addition, resveratrol enhances T-cell-mediated antitumor immunity by promoting aberrant PD-L1 glycosylation and dimerization, thereby preventing PD-1/PD-L1 interactions and, consequently, increasing the sensitivity of aggressive cancer cells to T-cell-mediated cell death [176,177].

These observations highlight the importance of synergistic multimodal therapy for the effective induction of immunogenic cell death through the coordinated activation of macrophages and other immune cells. Activation of M1 macrophages is associated with increased NO production, which exerts direct cytotoxic effects on tumor cells and promotes apoptosis. M1 macrophages increase the activity of the iNOS enzyme, which catalyses the conversion of L-arginine to NO and citrulline, while M2 macrophages preferentially express Arg-1 enzyme, which competes with iNOS for L-arginine [83,178]. Functionally, M1 macrophages are proinflammatory. They inhibit the proliferation of tumor cells and destroy damaged tissues by secreting proinflammatory cytokines (IL-12 and IL-23), ROS and NO, and have high antigen-presenting abilities [83,178]. Within the TME, NO production is essential for the antitumor activity of CD8+ T-cells [179]. NO enhances the expression of death receptors, thereby amplifying their antitumor and cytotoxic activity [180]. The effects of NO in cancer have attracted attention after the observation that activated macrophages metabolize arginine to produce NO, which functions as a key effector molecule in mediating tumor cell cytotoxicity [83,95,179,180]. NO produced by NOS2-expressing myeloid cells acts together with CD8+ T-cells to eliminate tumor cells. Therefore, elucidating the role of NO in tumor immunity is crucial for the development of effective immunotherapies that target different aspects of NO metabolism. The biological effects of NO in tumors are context-dependent and determined by its concentration, duration of exposure, cell sensitivity, extracellular milieu, and tissue localization of iNOS [181,182,183]. While low-to-moderate NO levels promote angiogenesis, invasion and metastasis and inhibit apoptotic processes, while higher NO concentrations exert cytotoxic effects and promote apoptosis [184]. Our data are consistent with these reports [95,179,180,181,182,183,184]. Specifically, we observed the polarization of TAM macrophages towards the M1 phenotype in all CP-treated groups (CP, CP + HT, Res + CP, and Res + CP + HT), together with reduced arginase activity. Increased iNOS levels were detected in all treated groups, especially in the Res, CP + HT, CP and Res + HT groups. Importantly, iNOS overexpression in TAMs has been associated with favorable prognostic outcomes in breast and lung cancers, high levels of apoptosis, and reduced disease recurrence after therapy [95,179,180,181,182,183,184]. The data are also consistent with the analysis of NO in splenic macrophages, demonstrating that resveratrol exerts immunomodulatory effects by stimulating NO production under basal conditions, while displaying anti-inflammatory properties in LPS-stimulated macrophages. Furthermore, treatment with CP, CP + HT and Res + CP enhanced NO production under inflammatory conditions, confirming polarization toward the M1 phenotype. On the other hand, arginase activity remained largely unchanged across all groups, although a slight decrease was observed following CP and Res + CP + HT treatment.

In contrast to M1 macrophages, M2 macrophages have anti-inflammatory and immunosuppressive properties, possess limited antigen-presenting capacity, and promote tumor growth and angiogenesis [185]. A growing body of evidence indicates that arginases 1 and 2 play a key role in the regulation of tumor growth and metastasis through various mechanisms, including the modulation of L-arginine metabolism, signaling pathways, and the TME. Therefore, arginase represents an attractive target for cancer therapy. Arginase-mediated immune escape of cancer cells occurs through several mechanisms: (i) elevated expression of Arg-1 and Arg-2 directly impairs T-cell function by depleting L-arginine in the TME; (ii) secretion of Arg-1 or release of extracellular vesicles containing Arg-1 from myeloid-derived suppressor cells (MDSCs) and TAMs reduces L-arginine availability in the TME, thereby suppressing T-cell activation and proliferation; (iii) increased Arg-2 expression in Treg cells enhances their suppressive capacity and accumulation through inhibition of mTOR signaling [186,187,188]. Consistent with these mechanisms, our results demonstrated a significant reduction in Arg-1 activity in the CP + HT and Res + CP + HT groups compared with controls. Decreased Arg-1 activity in these groups indicates the repolarization of M2 macrophages toward the M1 phenotype, resulting in enhanced antitumor efficacy and extended lifespan. Synergistic combinations of propolis, QU, naringenin or resveratrol with CP and HT further promote immunogenic tumor cell death through the induction of DAMPs, such as CRT, HSP70, and HMGB1, and by remodeling the TME via increased maturation of DCs and macrophages. Activated DCs and macrophages, as antigen-presenting cells, reactivate the effector function of cytotoxic T-cells on tumor cells, as well as the activation of NK cells via HSP70-dependent mechanisms [9,171,174], ultimately leading to the inhibition of tumor growth and prolonged lifespan.

Notably, resveratrol is widely recognized of as a dietary polyphenol with prominent chemopreventive and vasculoprotective properties. Aging is associated with a broad spectrum of changes, such as genomic instability, epigenetic remodeling, telomere shortening, loss of proteostasis, dysregulation of nutrient-sensing pathways, mitochondrial dysfunction, cellular senescence, and stem cell exhaustion. Various age-related and microenvironmental changes directly affect the vascular system, which is essential for maintaining tissue homeostasis through the delivery of oxygen and nutrients and the removal of metabolic waste. During aging, the density of blood vessels and the number of pericytes decline [189,190], accompanied with the reduced NO production and increased apoptosis of endothelial cells. Pericytes also serve as a source of fibroblasts during inflammatory processes, and their differentiation into fibroblasts becomes more pronounced with aging and in age-related diseases, including tumors. Cancer-associated fibroblasts (CAFs), along with other tumor-associated stromal cells, facilitate tumor progression and metastasis by promoting immunosuppression. In addition, physiological aging is characterized by a progressive impairment of immune surveillance, often accompanied with elevated expression of PD-L1, which promotes tumor initiation and progression. Resveratrol counteracts several of these processes by inhibiting the ROS-NF-κB axis, suppressing platelet activation, increasing NO bioavailability, and activating sirtuin pathways, thereby contributing to the preservation of a youthful vascular phenotype and delaying cellular senescence [191]. In addition, resveratrol functions as a natural inhibitor of the PD-1/PD-L1 immune checkpoint. It can directly block the N-linked glycosylation of nascent PD-L1 within the ER or directly bind PD-L1 at the cell surface, inducing its dimerization and blocking PD-1 binding. Through the suppression of PD-L1 signaling, resveratrol may exert immunometabolic effects that mitigate immune dysfunction and reduce cancer risk during aging [176]. Resveratrol-mediated HIF inhibition appears to prevent resistance to antiangiogenic therapies, as well as block the EMT and differentiation of myeloid suppressor cells into M2-proangiogenic TAMs. These effects contribute to the reactivation of NK and T-cell activity, prevent genetic instability in tumor endothelial cells, and reduce the selection of invasive, metastatic tumor cell clones that are resistant to antiangiogenic compounds [157,189,192]. The indirect effect of resveratrol on angiogenesis is based on a dose-dependent increase in p53 activity in cancerous and non-cancerous cells through the upregulation of antiangiogenic factors, including thrombospondin-1, and the reduction in VEGF expression in cancer cells [13].

In addition to resveratrol, flavonoids such as QU, kaempferol, apigenin or ginkgentin have been identified as inhibitors of PD-1/PD-L1 interactions. By inhibiting the PD-1/PD-L1 axis, flavonoids promote the cytotoxic activity of T-cells against cancer cells and strengthen antitumor activity by increasing the expression of immune markers such as CD8, granzyme B (GZMB), and IFN-γ. Disruption of the PD-1/PD-L1 axis by flavonoids promotes T-cell activity against cancer cells and suppress tumor growth [193,194,195]. These data highlight the potential of flavonoids in the development of novel modulators targeting the PD-1/PD-L1 pathway. Inhibition of the PD-1/PD-L1 axis is an important strategy to overcome the adaptive immune resistance of cancer cells, as cancer cells exploit this axis to induce immune escape in cancer development and progression. Immune checkpoint inhibitors (ICIs) directed against PD-1 or PD-L1 are increasingly used as standard therapy for various malignancies. Numerous studies have revealed high PD-L1 expression in lung cancer, melanoma, glioma, breast cancer and other malignant tumors, contributing to the formation of the immunosuppressive TME [196,197]. Based on binding affinities, Li et al. [198] evaluated a range of natural compounds, including kaempferol, cosmosin, tannic acid, pentagalloyl glucose, ellagic acid, resveratrol, urolithin A, and rifubutin, and categorized them into four groups based on their ability to block PD1/PD1L signaling: (i) compounds binding to both PD-1 and PD-L1; (ii) compounds selectively targeting the PD-L1 protein; (iii) compounds without binding ability; and (iv) binders without a functional blocking effect. Thus, the use of natural compounds to disrupt PD-1/PD-L1 interactions has emerged as a promising strategy for cancer immunotherapy. This approach may help to overcome several limitations related to antibody-based PD-1 inhibitors (e.g., pidilizumab and nivolumab) and PD-L1 inhibitors (e.g., atezolizumab and durvalumab), including off-target toxicity, poor permeability (to tumor tissues) and immunogenicity, as well as high production costs and challenges in quality control [198,199,200].

### 4.9. Nanoparticles: Characterization and Application in Cancer Therapy with Hyperthermal Treatment

Nanoparticles possess distinctive physicochemical characteristics, such as enhanced colloidal stability, adaptive surface functionalization, and a high surface-to-volume ratio, which make them particularly suitable for the targeted induction of localized HT in malignant tissues. Recent advances in nanotechnology have facilitated highly precise drug delivery to tumor sites, thereby reducing off-target toxicity, potentiating the therapeutic efficacy of RT, and improving the pharmacokinetics and bioavailability of anticancer agents [48,55,111,112,148,167]. Collectively, these innovative features establish nanotechnology as a powerful and versatile platform in modern oncology, particularly within the context of precision medicine.

Flavonoids exert antitumor activity through multiple mechanisms, influencing various stages of carcinogenesis while simultaneously offering cytoprotective effects on normal cells [83]. Despite these advantages, their clinical translation is limited by unfavorable physicochemical properties, including poor aqueous solubility and instability, as well as pharmacokinetic limitations such as rapid metabolism, short half-life, low bioavailability, and rapid elimination [43]. One promising strategy involves increasing the concentration of bioavailable flavonoids at the tumor site, thereby bypassing the rapid degradation in the gastrointestinal tract and liver [201,202]. Consequently, recent research efforts have focused on the development of novel flavonoid formulations that enhance the stability, solubility and bioavailability while enabling the controlled release and improved targeting of malignant cells.

The emergence of nanodrug formulations has significantly increased the therapeutic potential of flavonoids in oncology [48,55,147,201,202,203]. A wide range of nanoparticles have been investigated as carriers of flavonoids, either alone or in combination with conventional chemotherapeutic agents. Based on their improved absorption and enhanced targeting capabilities, organic nanoparticles, such as solid lipid nanoparticles, protein-based nanoparticles, and liposomes, as well as inorganic nanoparticles, including metal-bases and silicon dioxide nanoparticles, are increasingly employed in cancer drug delivery strategies [48,55,148,201,202,203].

There are two principal approaches in nanoparticle-mediated drug delivery. Passive targeting relies on the enhanced permeability and retention (EPR) effect, where nanoparticles preferentially accumulate within the tumor area due to the increased permeability of tumor blood vessels and inefficient lymphatic drainage. On the other hand, active targeting is achieved by functionalizing the nanoparticle surface with ligands that specifically recognize receptors on tumor cells. These ligand–receptor interactions facilitate cellular recognition, binding and internalization, minimizing drug leaking into the surrounding environment compared to passively targeted systems [204].

Numerous studies indicate that nanomedicine-based approaches in cancer treatment offer several advantages over conventional cancer therapies. These include: (i) improved site-specific delivery and controlled release; (ii) enhanced intratumoral drug accumulation and favorable pharmacokinetic properties due to the “EPR effect”; (iii) reduced effective therapeutic doses; (iv) increased drug bioavailability and stability; (v) improved cellular uptake; (vi) reduced systemic toxicity and adverse effects; (vii) enhanced therapeutic efficacy and specificity due to tumor-selective accumulation; (viii) the possibility of combination therapies; (ix) modulation of the TME with the potential enhancement of antitumor immune responses [203].

Nanosystems may exert their therapeutic effects through multiple mechanisms, including drug release in close proximity to tumor cells; surface binding to tumor cells and subsequent depot-like drug release, or direct cellular internalization via endocytosis.

Although inorganic and metallic nanoparticles are increasingly investigated in clinical settings due to their unique physiochemical properties, polymeric, liposomal, and nanocrystal-based systems dominate the cancer nanotherapy [205]. Polymeric nanoparticles are often composed of specific biocompatible materials, such as polyglycolic acid (PGA) and polylactic acid (PLA), which show preferential affinity for malignant cells. Nanocrystals are particularly advantageous due to their high loading capacity, stability, prolonged drug release, and capacity to deliver poorly water-soluble compounds.

Taken together, nanoformulated flavonoids and chemotherapeutic agents can potentially enhance anticancer efficacy. By counteracting the immunosuppressive effects of the TME on immune cells, improving antiangiogenic activity, and simultaneously exerting cytotoxic effects on tumor cells and their microenvironment, these nanosystems may ultimately lead to improved therapeutic outcomes [201,202,203,204,205,206].

The rapid proliferation of tumor cells promotes the development of aberrant, fenestrated blood vessels that facilitate the extravasation of macromolecules, including nanoparticles. At the same time, reduced lymphatic drainage within tumor tissue results in the prolonged retention of nanoparticles, leading to intratumoral concentrations that are approximately 10–50-fold higher than those observed in healthy tissue [201,202,203,204,205,206].

Consequently, nanoparticle size, charge, and surface properties are critical determinants of effective tumor accumulation via the EPR effect [207]. The delivery efficiency of nano-sized agents is dependent on several tumor-specific parameters that influence the EPR effect, including: (i) regional blood flow to the tumor, (ii) vascular permeability, (iii) structural barriers imposed by perivascular tumor cells and the extracellular matrix, and (iv) intratumoral pressure. Modulation of these factors can amplify the EPR effect, thereby improving nanoparticle delivery and enhancing anticancer efficacy. In general, nanoscale agents with molecular weights exceeding 50 kDa preferentially accumulate within tumors due to the EPR effect, followed by localized release of their therapeutic payloads. A wide range of biological and chemical mediators, including bradykinin, NO, peroxynitrite, prostaglandins, angiotensin-converting enzyme inhibitors, TNF-α, HO-1 and matrix-degrading enzymes such as collagenase or hyaluronidase, as well as VEGF and various cytokines, can normalize and/or increase blood pressure in vessels, leading to a short-term beneficial effect on tumor perfusion. In addition, physical interventions, such as ultrasound, ionizing radiation, heat, and photoimmunotherapy, can alter tumor vasculature and increase the nanosystem permeability and retention [201,202,203,204,205,206,207,208].

Nanoparticle bioavailability and half-life are influenced by surface characteristics that effectively overcome epithelial fenestration [209]. To prevent particle aggregation and premature clearance, polymeric coatings provide electrostatic repulsion and steric effects. The most studied coatings are made from polymers such as PLA, polyvinyl alcohol (PVA), polyethylene glycol (PEG), and polymethyl methacrylate (PMMA) [210].

Kučan et al. [13] investigated the antitumor potential of resveratrol nanocrystals in combination with CP in mice bearing solid EAT and exposed to whole-body HT. Resveratrol nanocrystals were administered orally via gastric cannula. The combined treatment regimens, CP + HT, nanocrystalline (NC) Res + CP, and NC Res + CP + HT, have shown pronounced antitumor effects, as evidenced by reduced tumor volume, tumor growth inhibition, and prolonged survival. The increase in lifespan was attributed to reduced nutrient availability in the digestive system, decreased expression of the GLUT-1 glucose transporter, diminished glucose uptake by tumor cells, and delayed tumor cell proliferation and growth.

Resveratrol has been shown to suppress lactate production, thus reducing tumor invasiveness and resistance to apoptosis. Specifically, lactate accumulation lowers intracellular pH, creating a metabolic advantage for tumor invasion, while the glycolytic pathway contributes to apoptosis resistance [83]. Resveratrol nanocrystals exerted more pronounced inhibitory effect than resveratrol, significantly suppressing glucose metabolism in cancer cells. Reduced glucose uptake subsequently limits glycolytic metabolism and, consequently, lactate production.

The body weight loss observed in treated mice, especially in the NC Res + CP + HT group, may be explained by altered nutrient absorption due to nanocrystal retention in the intestinal epithelium and mesenteric lymph nodes [211]. In contrast, in control mice bearing solid EAT, a significant increase in body weight was observed due to rapid and uncontrolled tumor growth.

The best survival improvement (% ILS; increased lifespan) was observed in the NC Res + CP (81.66%) and CP + HT (67.90%) groups, indicating the involvement of both direct cytotoxic effects and immunomodulatory mechanisms. Resveratrol can overcome MDR, and in combination with CP, it exerts additive and/or synergistic effects, enhancing the chemosensitivity of cancer cells. Moreover, resveratrol reduces cytotoxic effects on healthy cells and modulates immune responses within the TME by regulating cytokine/chemokine secretion, immune checkpoint signaling (MHC-I and PD-1/PD-L1), and key signaling pathways such as NF-κB and AKT/mTOR, as well as by promoting the activation of NK cell receptors [59,157].

NC Res may potentiate immune responses due to the improved bioavailability, thereby improving antitumor efficacy. Both resveratrol and its nanocrystals appear to modulate the PD-1/PD-L1 axis in cancer cells, supporting their potential use as adjuvants in combination with PD-L1 or anti-PD-1 inhibitors. These findings emphasize the importance of synergistic, multimodal treatment strategies capable of inducing immunogenic cell death through the coordinated activation of macrophages and other immune cells. From this perspective, NC Res combined with CP or other chemotherapeutic agents may significantly enhance therapeutic efficacy of anti-PD-1/anti-PD-L1 immunotherapies [212].

Beyond resveratrol, other polyphenols, such as naringenin (NAR), have demonstrated marked anticancer potential when formulated in nanoscale delivery systems. Encapsulation of NAR with various natural and synthetic polymeric materials, including chitosan, sodium alginate, HAP, PLGA, PVA and β-cyclodextrin, significantly improves its solubility, bioavailability, and plasma stability, thereby strengthening its anticancer efficacy [213,214].

Nagarajan et al. [215] developed a dual-responsive nanosystem consisting of polyacrylamide (PAM) encapsulated NAR loaded into magnetic iron oxide–mesoporous silica (Si) nanoparticles (Fe/Si-NAR/PAM) for synergistic chemo-HT treatment of non-small-cell lung cancer. This smart nanoplatform enabled controlled drug release and better therapeutic effects while reducing drug toxicity. Magnetic Hyperthermia (MHT), a promising adjunct cancer therapy, relies on the localized heat generated by magnetic nanoparticles, increasing tumor temperatures to 40–43 °C and inducing cancer cell death [215,216]. Due to the poor vascularization of tumor tissue, HT exacerbates hypoxia, lactic acid accumulation, low pH, and nutrient deficiency in tumor tissues, ultimately causing cancer cell death. The cytoprotective effects of NAR and its nanoformulations against oxidative stress are closely associated with the modulation of multiple signaling pathways, including Nrf2-HO-1, NO/cGMP/potassium channel signaling, COX-2, NF-κB, AMPK/SIRT3, PI3K/Akt/mTOR, BDNF, NOX, and LOX-1 pathways. A deeper understanding of the mechanisms behind the protective effects of NAR may facilitate the development of novel therapeutic strategies for oxidative stress-related diseases and cancer [216].

Among emerging nanomaterials, graphene has attracted considerable attention due to its remarkable physicochemical properties. Graphene and its derivatives are rich in functional groups, such as carboxylic and hydroxyl groups, which facilitate its surface modifications. Various anticancer biomolecules, such as siRNA, DNA and small-molecule drugs, can be loaded onto the graphene surface, facilitating applications in gene silencing, drug delivery and many others. Graphene-based strategies in oncology are based on (i) targeted drug delivery, (ii) photothermal therapy (PTT), (iii) photodynamic therapy, and (iv) imaging [217,218].

Metallic nanoparticles have also gained significant interest over the past two decades, especially in the form of metal–polyphenol complexes. These complexes increase the solubility, stability and biological properties of polyphenols, such as curcumin and resveratrol, potentially modulating metal toxicity. Metals typically chelate polyphenols like curcumin via their keto-enol groups, thereby improving their capability to act as photosensitizers under visible light.

Due to the unique properties of metals (copper, zinc, ruthenium, palladium, gallium, nickel, platinum, iron oxide, etc.), metal–curcumin complexes have been successfully applied in cancer diagnosis, therapy and phototherapy [219,220]. Metal–curcumin and metal–resveratrol complexes offer some advantages in tumor therapy, including (i) the delivery of one or more therapeutic drugs with improved bioavailability, active targeting tumor cell/tissue and/or response to the complex tumor environment for better drug penetration and sustained release; (ii) the possibility of using the same system to target tumor cells not only with chemotherapy but also with other therapeutic strategies, such as photodynamic therapy, drug delivery systems, and PTT, as well as optical imaging; and (iii) the development of environmentally friendly pharmaceutical platforms. Metallic nanoparticles such as gold nanoparticles (AuNPs) have emerged as promising candidates for cancer diagnosis and treatment [83,84,85,86,87,88,89,90] because of their unique optical, physical, and surface properties, together with their safety profile and biocompatibility, making them valuable tools in biomedical and pharmaceutical applications (see the review paper by [221]).

Among nanoaggregates, FerH represents simple but effective system formed through the coordination of a natural polyphenol, hematoxylin (HMT), and Fe^3+^ ions released from a commercially available iron supplement. The discrete FerH nanocomplexes are sufficiently small to penetrate the heterogeneous microstructures of solid tumors. Once within the tumor tissue, they undergo an in vivo pseudo-stepwise kinetic self-assembly process, resulting in the formation of interconnected nanoaggregates. This process is driven by the rapid coordination between polyphenolic ligands and metal ions, yielding metal–phenol networks (MPNs) that further assemble into network aggregates or multilevel assemblies.

Phototermal therapy exploits localized HT generated by photothermal agents (PTAs) and near-infrared (NIR; 700–900 nm) irradiation to induce irreversible thermal damage and the rapid ablation of tumor cells [222]. In this context, He et al. [222] evaluated an in vivo effect of PTA assembled from HMT, isolated from *Haematoxylum campechianum*, and Fe^3+^ ions supplied by the iron supplementation Ferrlecit™. Coordination between HMT and iron ions leads to the formation of FerH nanocomplex seeds, enabling deep tumor penetration prior to aggregation. FerH nanoaggregates exhibit high photothermal conversion efficiency in the NIR range compared with previously reported metal–phenolic materials. In addition, under weakly acidic TME conditions, FerH can be activated to catalyze the reaction between H_2_O_2_ and iron ions, generating highly cytotoxic hydroxyl radicals (•OH).

Due to their deep tumor penetration and prolonged intratumoral retention, FerH nanoaggregates enable the efficient elimination of primary tumors following a single-dose administration combined with upward laser irradiation. In addition to their direct cytotoxic effects, FerH-mediated PTT also triggers a robust immunogenic response. When combined with immune checkpoint agents that target the programmed cell death ligand-1 (anti-PD-L1), this strategy results in remarkable inhibition of distant, untreated tumors. DC maturation was significantly increased (58.5%), while the relative expression of CD80 and CD86 genes was increased by 1.8-fold and 1.9-fold, respectively, compared with anti-PD-L1 monotherapy. In accordance with enhanced immune activation, FerH-mediated PTT upregulated cytokine levels of IL-6 and TNF-α by 2.9-fold and 2.5-fold, respectively.

In addition to iron-based systems, a wide range of metals, including copper, zinc, ruthenium, palladium, gallium, nickel, and platinum, can be utilized in metal–polyphenol complexes for anticancer applications. In the presence of these metal complexes, HT can substantially enhance antitumor efficacy by directly inducing tumor cell death. Depending on their composition and structure, these complexes may function either as photothermal agents that convert light energy into heat or as catalysts for Fenton-type reactions, generating both heat and ROS. These combined effects can trigger various forms of regulated cell death, including apoptosis, necrosis, ferroptosis and cuproptosis, while simultaneously modifying the TME through diverse immunomodulatory mechanisms [223].

In addition to flavonoid-based nanoformulations, numerous conventional chemotherapeutic agents are now being used as nanocarriers to reduce systemic toxicity and improve therapeutic outcomes. Several clinically approved anticancer drugs have been developed as liposomal formulations, including etoposide (ETP), DOX, paclitaxel (PTX), irinotecan (IRI), erlotinib, docetaxel (DTX), vinorelbine (VNB), CP and epirubicin [224]. In addition, the antimetastatic efficacy of DOX-loaded liposomes conjugated with tretinoin has been investigated in cancers prone to lung metastasis, such as melanoma and breast cancer [224].

#### 4.9.1. Epigenetic Modulation and Antitumor Mechanisms of Resveratrol Nanocrystals

The inhibition of tumor growth and the improvement in survival outcomes may, at least in part, be attributed to the epigenetic effects of NC Res, particularly their modulation of HDAC activity. HDACs play a central role in tumorigenesis, regulating processes ranging from tumor initiation and progression to angiogenesis, metastasis, and the regulation of HSPs, HIF-1α, and other important cellular processes [225,226].

Adaptation to a hypoxic microenvironment is critical for tumor survival and metastatic spread. HIF-1α is a key mediator of this adaptation, promoting the production of proangiogenic factors and inducing enzymes involved in anaerobic metabolism. HDAC inhibitors markedly suppress HIF-1α expression and are currently in clinical evaluation, in part due to their potent antiangiogenic properties.

NC Res significantly modulate HDAC activity, either alone or in combination with CP and/or HT. Compared with conventional resveratrol, NC Res shows a more pronounced inhibitory effect on HDACs, thereby contributing more effectively to the suppression of tumor growth and progression [227]. As a HDAC inhibitor, NC Res demonstrates antiangiogenic activity by regulating the expression of proangiogenic genes, including VEGF, bFGF, and HIF-1α [228]. Numerous nanoformulations of natural flavonoids and their derivatives have similarly been shown to inhibit HIF-1 signaling and disrupt critical glycolytic components in cancer cells, including pyruvate kinase M2(PKM2), lactate dehydrogenase (LDHA), GLUTs, hexokinase II (HKII), phosphofructokinase-1 (PFK-1), and pyruvate dehydrogenase kinase (PDK).

HDAC inhibition can further impair DNA repair mechanisms, thereby sensitizing tumor cells to chemotherapy and RT by enhancing the DNA-damaging effects of these treatments [229]. Excessive HDAC activity is known to result in the aberrant repression of tumor suppressor genes [230], while hypoxic conditions can modulate HDAC function both directly and indirectly through the HDAC-mediated regulation of oxygen-dependent gene expression and hypoxia-induced angiogenesis [231]. In addition, NC Res effectively inhibit HSP70 activity [13].

Nanomedicine-based tumor therapies offer certain advantages compared to conventional treatments, including improved drug delivery and controlled release at targeted sites, enhanced tumor accumulation, and favorable improved pharmacokinetic properties due to the “EPR effect” [201,202,203,204]. These properties may potentiate the antiangiogenic effect of NC Res while alleviating suppressive effect of the TME on immune cells, ultimately enhancing the cytotoxic effects on tumor cells and improving therapeutic outcomes [232]. The abnormal tumor vasculature, characterized by fenestrated and poorly organized blood vessels, as well as reduced lymphatic drainage, facilitates nanoparticle extravasation and retention. As a result, nanoparticle concentrations within tumors can be 10–50 times higher than in healthy tissues [233,234].

These mechanisms likely contribute to the enhanced antitumor efficacy observed with NC Res in combination with CP. HT can further enhance the cytotoxic effect of CP on tumor cells, promote tumor cell death and, together with NC Res, strengthen the immune response. This combined approach may reduce tumor resistance to chemotherapy and increase the lifespan in experimental models. Furthermore, the HDAC-inhibitory activity of resveratrol nanocrystals may suppress tumor growth and overcome chemoresistance by modulating immune checkpoint pathways, enhancing immune surveillance, and increasing tumor sensitivity to both chemotherapy and immune checkpoint inhibitors. However, treatment with NC Res combined with CP, with or without HT, increases the level of HSP70.

It is also interesting that, following recovery at physiological temperature (37 °C), HSP expression remains only in tumor cells but not in normal cells [1]. Cells with enhanced HSP expression are particularly sensitive to the cytotoxic effects of NK cells. Moreover, during tumor cell necrosis, HSPs are released in the extracellular milieu, stimulating macrophages and dendritic cells to secrete cytokines and activate antigen-presenting pathways, ultimately strengthening antitumor immune responses [235]. These findings indicate that macrophage activation can be driven not only by NC Res but also by apoptosis, HT, and HSPs. Consequently, HSP70 plays a dual role in tumor biology: in addition to its involvement in inducing apoptosis, it exerts a pronounced immunostimulatory function.

#### 4.9.2. Flavonoids as Nanoinhibitors in Overcoming HSP Expression

A carrier-free bifunctional nanoinhibitor was developed to overcome HSP-mediated resistance during HIPEC. The nanoinhibitor was formed by controlled coordination between manganese ions (Mn^2+^) and EGCG. The resulting nanoinhibitor directly inhibits HSP90 by depleting intracellular ATP levels, while the combined effects of HT and Mn^2+^ ions synergistically promote oxidative stress, caspase-1 expression, and tumor cell death by pyroptosis. This form of inflammatory programmed cell death enhances tumor immunogenicity by facilitating antigen release and dendritic cell maturation. This strategy of inhibiting heat resistance in HIPEC turns immunologically “cold” tumors into “hot” ones, leading to the eradication of disseminated abdominal tumors and the induction of a robust immune response. This nanoinhibitor EGCG effectively induces pyroptosis in colon cancer cells, inhibiting resistance to heat stress and increasing oxidative stress, which provides a new strategy for the treatment of colorectal peritoneal metastases.

Importantly, EGCG has already entered into phase I and II clinical trials for the treatment of various cancers, including colorectal, prostate, lung, breast, esophageal, bladder, pancreatic (NCT02336087), superficial skin (NCT02029352), and urothelial (NCT01993966) tumors, indicating its translational potential for cancer treatment [236,237].

#### 4.9.3. Modulated Electro-Hyperthermia (mEHT) with Flavonoids

Modulated electro-hyperthermia (mEHT) is an emerging non-invasive adjuvant therapy that applies a modulated electromagnetic field to tumors, inducing damage to tumor cells through both temperature-dependent and temperature-independent mechanisms [238]. Thermal effects include cell cycle arrest, hypoxia, acidosis, protein denaturation, and altered blood perfusion, while non-thermal effects involve electroporation, immunomodulation, direct DNA damage, inhibition of angiogenesis, and modulation of bioelectrical signals [238].

mEHT has been shown to enhance drug uptake in tumor cells, thereby increasing the cytotoxicity of chemotherapeutics [239]. In clinical settings, mEHT has demonstrated therapeutic benefits in patients with breast [240], cervical [241], ovarian [242], rectal [243], and pancreatic cancers [244,245]. Electromagnetic field exposure can also induce DNA damage in cancer cells via several mechanisms, including the generation of ROS and disruption of DNA repair pathways, leading to genomic instability and cell death. Additionally, mEHT may inhibit angiogenesis by downregulating VEGF production, as observed in breast cancer cells [246], likely through interference with bioelectrical signals essential for new blood vessel formation.

By triggering an immune response, mEHT helps the immune system to recognize and attack cancer tumor cells, shifting the balance toward tumor suppression [247] through the induction of immunogenic cell death and modification of the TME [248]. This process recruits and activates immune effector cells, including dendritic cells [249], cytotoxic T-lymphocytes [250], and NK cells [251]. Moreover, mEHT may act synergistically with immune checkpoint inhibitors, further enhancing the antitumor immunity and inducing abscopal effects [252,253,254].

Kuo et al. [255] demonstrated that combining mEHT with curcumin and resveratrol increases immune cell infiltration into the CT26 tumor, where the overexpression of HSP70 mediates the recruitment of APCs, resulting in enhanced antitumor efficacy [255]. Resveratrol in combination with mEHT exerts its anticancer effects by inhibiting Akt phosphorylation, leading to the suppression of HSF1 activation in cancer cells and, consequently, the repression of tumor growth. Resveratrol also inhibits HSF1 nuclear translocation, thereby downregulating HSP70 expression [255]. Co-treatment with mEHT and nanoformulations of curcumin and resveratrol significantly induced cell cycle arrest and apoptosis in CT26 cells. Mild HT treatment with curcumin and resveratrol synergistically activate host immunity and inhibit cancer development with limited side effects. Serum concentrations of curcumin and resveratrol were significantly increased during mEHT application, promoting apoptosis, HSP70 expression in tumor cells, and recruitment of CD3^+^ T-cells and F4/80^+^ macrophages [255].

Similarly, Chakraborty et al. [256] reported that curcumin with mEHT inhibiting HSF1 nuclear translocation suppresses its transcriptional activity and reduces HSP70 upregulation. Curcumin additionally upregulates HLJ1, a heat shock tumor suppressor protein, which inhibits invasion and metastasis in lung cancer cells [257] while reducing HSF1 expression and the proliferation of oral squamous carcinoma cells [258]. These findings highlight the potential of HSF1 inhibitors to enhance tumor therapy and warrant further preclinical studies [256,257,258]. Viana and Hamar [238] also explored small-molecule inhibitors such as KRIBB11 to downregulate the heat shock response (HSR) and augment tumor cell death. In vitro studies have shown that combining QU and KRIBB11, two potent heat shock inhibitors, with mEHT reduces breast cancer cell viability and suppresses the HSP70 mRNA upregulation typically observed with mEHT monotherapy [259]. The combination of mEHT and KRIBB11 also decreases heat shock-associated complement production via C4b, an acute-phase protein [238]. QU competitively binds to HSEs [260], enhancing HSP70 accumulation in tumors and facilitating apoptosis through the HSF1 pathway [261]. However, QU appears to inhibit multiple targets beyond HSF1, including protein kinases, suggesting a nonspecific mechanism of action [262]. Current evidence suggests multiple mechanisms underlying HSF1 inhibition: (i) inhibition of HSF1 phosphorylation, thereby preventing its activation; (ii) prevention of HSF1 nuclear translocation (e.g., by dorsomorphin and resveratrol); (iii) inhibition of HSF1 binding to HSEs (e.g., by QU, fisetin, and curcumin); (iv) blockade of HSF1-dependent recruitment of the positive transcription elongation factor (p-TEFb), thereby disrupting downstream effects (e.g., by KRIBB11 and cantharidin); (v) impairment of HSF1 function through CDK9 inhibition; and (vi) suppression of HSF1-mediated transcriptional activity [238]. Therefore, downregulation of the HSR, either through direct HSF1 gene downregulation or HSF1 pharmacological inhibitors, represents a promising strategy to enhance the cytotoxic and immunogenic effects of mEHT in tumor therapy.

## 5. Hyperthermia, Radiation, and Flavonoids

HT, radiation, and flavonoids show complex interaction in cancer therapy. In summary, HT enhances the therapeutic efficacy of RT and promotes antitumor immunity through multiple mechanisms: (i) combined RT and HT exacerbate hypoxia and reoxygenation, resulting in structural damage and the accumulation of lethal DNA lesions, which leads to cell cycle arrest or cell death; (ii) HT potentiates radiation-induced oxidative stress while simultaneously suppressing DNA damage repair and recovery; and (iii) the combination of RT and HT stimulates antitumor immune responses and activates immune cells. Flavonoids can function both as protective agents and as radiosensitizers. In normal cells, they act as antioxidants, protecting against radiation-induced damage by scavenging free radicals, reducing inflammation, and supporting DNA repair mechanisms. In contrast, in tumor cells, flavonoids enhance radiosensitivity by inhibiting cancer cell survival pathways, such as NF-κB signaling, inducing apoptosis (programmed cell death), and impairing DNA repair processes (see the review paper by [39]).

### 5.1. Immunomodulatory Effects of Hyperthermia in Combination with RT

As discussed earlier, HT induces the release of tumor antigens from cancer cells, thereby enabling the immune system to recognize and target malignant cells more effectively [263]. The combination of RT with HT can enhance antitumor immune responses by promoting the release of antigens and cytokines (e.g., HSP70 and HMGB1) and by increasing the activity and abundance of immune cells, such as NK cells, DCs, and T-cells [263].

HSP70 strengthens the immune response by binding to tumor-derived antigens and facilitating their delivery to antigen-presenting cells, thereby promoting their maturation and the release of proinflammatory cytokines (IL-8 and IL-12), the upregulation of the costimulatory protein CD80, and the expression of the chemokine receptor CCR7 by DCs and macrophages both in vitro and in vivo. DCs subsequently present these tumor antigens and stimulate cytotoxic CD8^+^ T cell responses [264].

Consistent with these findings, magnetic induction hyperthermia (MIH) in a murine 4T1 metastatic breast cancer model significantly reduced primary tumor volume and lung metastases, improved survival, and increased Bax expression, as well as the proportion and ratio of CD4^+^/CD8^+^ T cells, compared with either RT or MIH alone [265]. MIH enhances the antitumor efficacy of RT by promoting Bax-mediated cell death, stimulating the immune activity of irradiated cells, and suppressing the RT-induced upregulation of MMP-9 [265].

Similarly, Werthmöller et al. demonstrated that HT (41.5 °C and 60 min) combined with RT (2 Gy) elicited a stronger immunogenic response than RT alone, resulting in a pronounced antitumor effect in radioresistant B16-F10 melanoma cells derived from C57BL/6 mice. The combined RT + HT treatment enhanced apoptosis, necrosis, the release of HMGB1 and HSP70, and the infiltration of CD8^+^ T cells, DCs, and NK cells [266].

In agreement with these observations, Mahmood et al. found that HT (42.5 °C and 30 min) combined with RT (8 Gy) effectively reduced tumor volume and increased the number of cytotoxic CD8α^+^ T cells in C57BL/6 mice injected with Panc02 cells [53]. Moreover, the combination of RT, HT, and immunotherapy further increased the number of CD4^+^ T-cells, suggesting a synergistic enhancement of antitumor immune responses.

### 5.2. Role of Flavonoids and Hyperthermal Treatment in Radiosensitization and Immunomodulation

Flavonoids can act both as radioprotectors and radiosensitizers. As radioprotectors, they upregulate antioxidant enzymes and enhance DNA repair processes in damaged cells. As radiosensitizers, flavonoids inhibit pro-survival pathways, such as NF-κB and Akt, while promoting apoptotic signaling [39]. Cancer cells have a higher demand for metals to sustain continuous proliferation, and flavonoid–metal interactions are critical for their radiosensitizing activity.

Flavonoids enhance the radiosensitivity of cancer cells through multiple molecular mechanisms. Radiation activates the PI3K/Akt, ERK, and N-κB pathways, which promote resistance by upregulating antiapoptotic and DNA repair proteins [267]. Flavonoids counteract these pathways, inducing apoptosis and cell cycle arrest. Their pro-oxidative properties further increase DNA damage and suppress DNA repair mechanisms [268,269].

Furthermore, flavonoids can undergo metal-catalyzed oxidation, generating free radicals and H_2_O_2_, which amplify radiation-induced oxidative stress and apoptosis [269,270,271]. Elevated intracellular Cu levels in cancer cells facilitate this process, enhancing ROS generation and promoting apoptotic cell death [268,269,272]. At lower concentrations, however, flavonoids may exhibit cytoprotective effects, emphasizing the importance of ROS production for their cytotoxic activity [268,269,272]. Moreover, Cu chelation by flavonoids disrupts Cu-dependent pro-carcinogenic processes, representing a promising therapeutic strategy, particularly because normal cells contain significantly lower metal ion levels [268,269,272].

Flavonoids enhance ionizing radiation (IR)-induced apoptosis through the inhibition of Akt signaling, ROS generation, and downregulation of the antiapoptotic protein Bcl-2 [273]. Several flavonoids have been reported to potentiate the effects of RT [59], while the combined application of flavonoids, RT, and HT can further augment radiosensitization by inhibiting DNA-PKcs and ATM, suppressing the NHEJ DNA repair pathway, and promoting mitotic catastrophe [59]. This enhanced tumor cell apoptosis is associated with increased PARP cleavage, activation of caspase-3, and reduced Bcl-2 expression. The combined treatment with flavonoids, RT, and HT also reduces cancer cell proliferation, metastasis, and angiogenesis, underscoring its potential as a multimodal therapeutic strategy for overcoming radioresistance [59].

HT inhibits the repair of DNA DSBs, which are major inducers of tumor cell death following IR. Combination therapies with PARP inhibitors such as flavonoids may further enhance the inhibition of DNA repair. Flavonoids like flavone, QU, fisetin and myricetin can inhibit PARP-1 activity. Oral PARP inhibitors have shown favorable therapeutic outcomes with acceptably low toxicity in breast cancer patients with the BRCA1 and BRCA2 mutation [274]. Flavonoids such as naringenin and naringin show similar PARP inhibitory effects and selectively induce cytotoxicity in BRCA2-deficient cells.

RT and HT also increase the expression of immune checkpoint molecules (ICMs), including PD-L1, PD-L2, and HVEM, compared with RT alone. This suggests that combination therapy with HT, RT and PD-1/PD-L1 immune checkpoint inhibitors may enhance antitumor immune responses in primary and metastatic tumors. Tumor immune evasion often involves the inactivation or suppression of effector T-cells via inhibitory receptors, such as programmed cell death protein-1 (PD-1), TIM-3 (mucin3) and LAG-3 (lymphocyte activation gene protein-3), and CTLA-4 [10,11]. Flavonoids like QU, apigenin, kaempferol, genistein, luteolin, hesperetin and axillarin can inhibit PD-1/PD-L1 signaling, promoting T-cell-mediated antitumor responses and cytokine production [275]. PD-1/PD-L1 signaling inhibits T-cell proliferation and cytokine production, and CTLA-4 competes with CD28 for binding to CD80/CD86, thereby reducing T-cell activation. LAG-3 binds MHC class II molecules, impairing antigen presentation and increasing the activity of Tregs, leading to immune suppression [13,275]. Flavonoids modulate these immune cells, shifting the TME from immunosuppressive toward immunostimulatory state. Nobiletin, QU, baicalein, apigenin, galangin and curcumin inhibit the expression of PD-L1 and reverse TME immunosuppression via the JAK/STAT and NF-κB pathways [13,39,83,276]. At the molecular level, flavonoids can affect PD-1/PD-L1 pathway by (i) the direct inhibition of PD-1 and PD-L1 expression; (ii) suppression of IFN-γ-induced upregulation of PD-L1; (iii) interference with the JAK/STAT/IFN regulatory factor 1 signaling pathway activated by IFN-γ or the EGFR/JAK2/STAT3 signaling pathway; (iv) direct targeting of EGFR at the cell membrane, thus inhibiting EGFR/STAT3/PD-L1 signaling; and (v) attenuation of STAT3 activation via the JAK1/JAK2/Src pathway and suppression of Myc activation through the Ras/RAF/MEK/ERK pathway [276,277,278].

Beyond immune checkpoint modulation, flavonoids exert immunomodulatory effects on various immune cells, including monocytes and macrophages, DCs, Tregs, MDSCs, NK cells and T-cells. Several mechanisms underlying the effects of propolis and its flavonoids on monocyte/macrophages function have been described: (i) flavonoids such as resveratrol, curcumin, QU, luteolin and catechins reverse immune suppression by inhibiting macrophage recruitment; (ii) flavonoids regulate TAM polarization by modulating key signaling pathways, including STAT3, NF-κB, Arg-1 and RelB/p52 signaling pathways and by promoting the IL-12-dependent recruitment of NK cells and cytotoxic T-lymphocytes to tumor sites; (iii) flavonoids enhance macrophage phagocytic activity either by inhibiting macrophage migration inhibitory factor (epicatechins), thereby enhancing anti-inflammatory and phagocytic functions [13,41,276,279,280], or by stimulating lysosomal degradation in macrophages [276].

Flavonoids also target MDSCs by suppressing the recruitment of immune-suppressive cells and decreasing MDSC-mediated immune suppression within the TME. In this context, flavonoids enhance the cytotoxic activity of T-cells and NK cells by producing Arg-1, iNOS and ROS. In a murine breast cancer model, EGCG significantly reduced the immunosuppressive activity of MDSCs by downregulating key components of the canonical signaling pathways involving Arg-1, iNOS, NADPH oxidase 2, NF-κB, and STAT3 [281]. Similar immunomodulatory effects have been reported for silymarin, icariin, and chrysin, which enhance CD8^+^ T-cell activity, promote the differentiation of MDSCs into immune-stimulatory macrophages and DCs, and suppress the expression of immunosuppressive mediators, including IL-10, IL-6, TGF-β and TNF-α. Furthermore, flavonoids reduce serum levels of immunosuppressive molecules, including TGF-β, prostaglandin E2 and COX-2, in tumor-bearing mice, thereby inhibiting Treg development and function, which indirectly limits tumor growth by reducing angiogenesis and regulating inflammation [276].

Furthermore, a specialized subset of DCs, often termed as killer DCs, has been shown to express various TNF family members, including Fas ligand (FasL), TNF-related apoptosis-inducing ligand (TRAIL) and TNF-α, enabling them to directly induce apoptosis in tumor cells [282,283]. Th1 lymphocytes further potentiate DC-mediated cytotoxicity via an IFN-γ-dependent pathway [284]. Kaempferol and QU increase the secretion of GM-CSF by PC-3 prostate cancer cells, thereby promoting the recruitment of DCs to the tumor site. In addition, some flavonoids enhance the immunogenicity of dying tumor cells by stimulating the release of calreticulin and HMGB-1, and by inducing expression of DC maturation markers, including CD80, CD86 and MHC II. Activated DCs subsequently secrete IL-12, which promotes the differentiation of naive CD4^+^ T-cells into Th1 cells and stimulates IFN-γ production, thereby reinforcing antitumor immunity [285].

Flavonoids also exert potent immunostimulatory effects on NK cells. Following flavonoid exposure, activated NK cells secrete cytokines such as IFN-γ and TNF-α. It has been shown that apigenin activates the JNK and ERK signaling pathways in NK cells, leading to the increased expression of perforin, granzyme B and the receptor natural killer group 2 member D (NKG2D), thereby boosting NK cell-mediated cytotoxicity against cancer cells [286]. Moreover, apigenin promotes the upregulation of other NK cell-activating receptors (NKp30 and NKp44), which enhances the surface expression of CD95L (FasL), resulting in the induction of apoptosis in hepatocellular carcinoma cells [117]. NK cells primarily induce tumor cells via death receptor-mediated pathways, such as Fas/FasL interactions [112], by recognizing cancer cells due to their decreased MHC class I expression.

Cytotoxic T-lymphocytes similarly activate the Fas/FasL pathway and secrete cytokines, including IFN-γ, to further enhance the antitumor immune responses. The antitumor activity of cytotoxic T-lymphocytes can also be increased by naringenin, which activates CD169^+^ macrophages in lymph nodes, thereby upregulating the expression of immune-associated genes such as CD169, IL-12 and CXCL10, leading to the recruitment of cytotoxic T-cells to tumor sites. In addition, hesperidin has been shown to stimulate Vδ1^+^ T-cells, a type of tissue-resident γδ T-cell that plays a role in both innate and adaptive immunity by recognizing cellular stress signals associated with viral infections and cancer. Collectively, flavonoids enhance the proliferation and effector functions of CD8^+^ T-cells, at least in part, through the downregulation of immunosuppressive mediators such as TGF-β and PD-L1. The coordinated activation of these immune cells ultimately contributes to prolonged survival in tumor-bearing animal models.

Nanoparticles can deliver flavonoids (like EGCG, QU, chrysin, curcumin, hesperidin and ferulic acid) directly to cancer cells, acting as heat mediators (e.g., gold nanoparticles and iron oxide), making them more sensitive to RT (using X-rays or light) or MHT (using alternating magnetic fields), thereby synergistically killing cancer cells [287,288,289,290,291]. MHT uses magnetic nanoparticles (MNPs) to generate localized heat (43–46 °C) in tumors, enhancing radiation/chemotherapy effectiveness by damaging DNA and increasing cell permeability, while flavonoids (like QU) have independent anticancer properties (antioxidant and apoptosis induction) and can be combined with MHT/radiation to target tumors or improve delivery, offering a multi-pronged, potentially less toxic approach to cancer treatment [289]. Tousi et al. [290] found that methoxypoly(ethylene oxide)-b-poly(lactide-co-glycolide (mPEG-b-PLGA)-coated iron oxide nanoparticles (IONs) loaded with the flavonoid eupatorin increased apoptosis and decreased necrosis in prostate cancer cells compared to free eupatorin or uncoated nanoparticles. Green iron nanoparticles (Rosemary-FeNPs), mainly containing phenolic acids, carnosol derivatives, and flavonoids, exerted greater cytotoxic effect than total extract on both 4T1 and C26 cancer cell lines. Many cancer cells are particularly sensitive to polyphenols, resveratrol, flavonoids or anthocyanins [267] present in natural extracts, which can trigger apoptosis or increase cell responsiveness to other treatments, such as chemotherapy, photodynamic therapy, radiation or HT [291].

### 5.3. Flavonoids Can Overcome Thermotolerance in Tumor Cells in Fractionated thermoRT

The development of thermotolerance in tumor cells is a major limitation in the treatment of cancer using fractionated thermoradiotherapy (thermoRT). Thermotolerance is primarily associated with the upregulation of HSP synthesis [292]. Although HSPs protect normal cells from general protein damage induced by heat shock, they also confer resistance to various stressors such as hypoxia, heavy metals, toxic chemicals, oxidative free radicals, glucose deprivation, and viral infection [293,294]. The transcription of heat shock genes is activated by heat shock transcription factors (HSFs) that bind to HSEs in the promoters of these genes. The transcriptional activity of HSFs is regulated by phosphorylation catalyzed by protein kinases [293,294]. Among these factors, HSF1 plays a central role in the development of thermotolerance and protection from heat-induced apoptosis.

Several mechanisms have been proposed to contribute to HT sensitization by QU and other flavonoids. These include: (i) the induction of intracellular acidification through the inhibition of lactate transport [95,143,295], (ii) inhibition of HSP70 synthesis [95,153,296], (iii) downregulation of HSF1 expression [153,270], (iv) inhibition of protein kinases involved in stress signaling [83,138,144], (v) and induction of apoptosis [9,95,133,138,139,140,141,142,143,144,297]. Compared to normal cells, the environment of tumor cells is acidic and tumor cells are adapted to grow at a low pH. QU is most effective at inhibiting thermotolerance under acidic conditions [144]. In this way, QU-mediated inhibition of thermotolerance not only enhances the response to HT but also improves responsiveness to other modalities of tumor therapy, including chemotherapy and radiation. It has been reported that QU inhibits the synthesis of all HSPs identified so far when cells are cultured at a low pH. The suppression of thermotolerance by QU may be attributed to its non-selective inhibition of protein kinase activity. For example, QU (100 μM) reduced total protein synthesis to 80% of control levels in Chinese hamster ovarian carcinoma cells (OvCa) at a pH of 6.7 compared with only a 30% reduction at a pH of 7.3 [144,296,297]. These findings suggest that proteins involved in heat shock-induced signaling pathways upstream of HSP expression, such as MAPKs, may contribute to the development of thermotolerance in cells adapted to growth at a low pH [144,298].

Given that HSP70 is a strong negative regulator of cell death and a critical factor supporting tumor cell survival and proliferation, its overexpression within tumors is associated with poor clinical outcomes. The depletion of HSP70 not only increases tumor cell death but also selectively sensitizes malignant cells to chemotherapeutic agents. Indeed, the downregulation of HSP70 reverses drug resistance in cancer cells, likely due to its ability to inhibit apoptosis both upstream and downstream of mitochondrial signaling pathways [95,153,296]. Furthermore, the inhibition of HSP90 often results in the compensatory overexpression of HSP70 [95,153,296], which can reduce apoptosis and diminish the antitumor frequency of HSP90 inhibitors [95,153,296]. Therefore, the simultaneous inhibition of HSP90 and HSP70 may significantly potentiate the antitumor and antiangiogenic efficacy of therapeutic agents [13]. Interestingly, numerous flavonoids, including the diosmin–hesperidin complex, exhibit antiangiogenic and radiosensitizing properties mediated through the inhibition of HDAC activity and the disruption of the HIF-1α–HSP-70/90 signaling axis, as demonstrated in an Ehrlich solid carcinoma (ESC) xenograft mouse model [299]. In addition, a number of chemotherapeutic agents display radiosensitizing activity and are capable of enhancing the efficacy of RT at primary tumor sites. Flavonoids, acting as HDAC inhibitors, contribute to their antitumor potency.

Previous studies have shown that HIF-1α degradation is mediated by its interactions with the von Hippel–Lindau (VHL) protein, a tumor suppressor that acts as an E3 ubiquitin ligase [300,301]. This mechanism is supported by high levels of HIF-1α expression and the high degree of vascularization observed in tumors lacking functional VHL. Kim et al. reported that HDAC inhibitors reduce HIF-1α levels by enhancing p53 and VHL activity, thereby promoting HIF-1α degradation. In contrast, Kong et al. [300] showed that HDAC inhibitors induce proteosomal degradation of HIF-1α through a mechanism that is independent of VHL, and that p53 and does not require the ubiquitin system. This alternative degradation pathway involves the increased interaction of HIF-1α with HSP70 and is secondary to the disruption of the HSP70/HSP90 axis. In this context, it appears that flavonoids, by inhibiting HDAC activity, suppress HIF-1α expression, disrupt the HSP70/90 stress response, and consequently downregulate the main downstream effectors, including the proangiogenic VEGF and pro-survival oncoproteins. Collectively, these effects result in tumor regression and increased survival.

## 6. Challenges and Future Trends

Accumulating experimental, preclinical, and clinical evidence demonstrates that HT is a highly effective sensitizer to both RT and cytotoxic chemotherapy in cancer treatment. When combined with chemotherapy or RT, HT can directly target hypoxic tumor cells and induce both direct and indirect cytotoxicity and apoptosis. In addition, HT increases chemo- and radiosensitization, improves tumor oxygenation, enhances cellular drug accumulation, inhibits DNA damage repair, strengthens antitumor immune responses, and induces profound alterations in the TME. Additional therapeutic effects mediated by yet unknown mechanisms have also been suggested. Consequently, HT is expected to play an increasingly important role in future cancer therapy by improving the efficacy of RT, chemotherapy, and immunomodulation targeting the TME [1,2,3,4,5,6,7,8,9,10,11,12,13].

HT can overcome tumor cell resistance to chemotherapy and RT while enabling dose reduction in these treatments, thus minimizing damage to surrounding healthy tissues. It enhances cancer cell cytotoxicity and stimulates antitumor immunity through the activation of immune cells, modulation of immune checkpoint pathways, induction of immunogenic cell death, and remodeling of the TME, particularly through the increased expression of HSPs. Furthermore, HT has been shown to reverse resistance to chemotherapeutic agents such as CP, doxorubicin, and paclitaxel by increasing drug uptake, altering tumor microcirculation, improving blood flow, modulating membrane permeability, and reprogramming cellular metabolism, underscoring its potent chemosensitizing capacity [13,14,15,16,17,18,19].

On the other hand, various polyphenolic compounds exhibit significant anticancer activity through numerous mechanisms. These include the induction of cell cycle arrest and inhibition of cancer cell proliferation, reduction in cancer stem cell populations, induction of apoptosis, modulation of oncogenic signaling cascades, inhibition of MMPs, suppression of invasion and metastasis, antiangiogenic and anti-inflammatory effects, and epigenetic regulation. Additional actions include the modulation of macrophage polarization, antiviral and antibacterial activities, regulation of glucose metabolism, and alteration of gut microbiota composition [9,10,13,39,41,43,83,95,133,138,139,236,281,299].

A multimodal therapeutic strategy combining natural compounds, especially in nanoformulated forms, with established cancer therapies exhibits synergistic antitumor effects through simultaneous actions on multiple biological levels:

1. Tumor cytotoxicity and apoptotic pathways
  − Induction of mitochondrial dysfunction and activation of intrinsic apoptotic pathways.
  − Enhancement of HT sensitivity through intracellular lactate accumulation and acidification, increased lysosomal activity, and downregulation of the HSF-1/HSP70 stress response, thereby intensifying heat-induced tumor cell death.
2. Drug accumulation and delivery
  − HT increases membrane permeability, tumor blood flow, and thermally induced membrane changes that facilitate drug uptake.
3. TME modulation and angiogenesis
  − Reduction in blood vessel density and attenuation of hypoxic gradients through inhibition of VEGF signaling.
  − Flavonoid-mediated remodeling of the TME, reduced perfusion, and increased hypoxia, enhancing intratumoral local drug retention and efficacy.
4. Immunomodulation
  − HT promotes T-cell infiltration, reduces regulatory T-cells, modulates immunosuppressive mediators, and enhances dendritic cell activation.
  − Flavonoids downregulate PD-L1 expression, modulate JAK-STAT pathways, and promote CD8^+^ T-cell-mediated antitumor responses.
  − Both modalities synergistically promote immunogenic cell death, improve antigen presentation, and enhance recruitment of cytotoxic immune effector cells.
5. Induction of danger-associated molecular patterns (DAMPs)
  − Upregulation of HSPs and other heat-induced proteins functioning as immunostimulatory signals.
  − Release of HMGB1 and surface exposure of calreticulin and HSP70, promoting immunogenic cell death.
6. Suppression of HSPs and thermotolerance
  − Flavonoids reduce the expression of key stress proteins, including HSP70 and HSP27, thereby diminishing thermotolerance.
  − Given the role of HSPs in immune recognition, their inhibition may further enhance antitumor immunity.
7. Reversal of drug resistance
  − Inhibition of MRPs and suppression of signaling pathways involved in survival and stress response, including NF-κB, STAT3, MAPK, AMPK, and HIF s, thereby restoring CP sensitivity.
8. Antiangiogenic and antiproliferative effects
  − Inhibition of angiogenesis and tumor cell proliferation through topoisomerase I/II inhibition and interactions with estrogen receptor β-induced signaling pathways.
9. Regulation of apoptosis-related gene expression
  − Downregulation of antiapoptotic genes (Bcl-2 and Bcl-xL) and upregulation of proapoptotic genes (Bax and PUMA), accompanied by activation of p53 and caspase cascades.
10. Oxidative stress and ROS modulation
  − HT increases intracellular ROS levels, contributing to cytotoxicity.
  − Flavonoids exert dual effects by enhancing ROS within tumor cells while protecting normal tissues from oxidative stress.
11. DNA damage and repair inhibition
  − HT inhibits DNA repair pathways (e.g., homologous recombination), potentiating the effects of DNA damaging agents.
  − Flavonoids may further inhibit repair mechanisms (e.g., ATM kinase activity), prolonging DNA damage.
12. Protection of normal tissues
  − Through antioxidant, anti-inflammatory and membrane-stabilizing properties, flavonoids such as QU protect renal and hepatic tissues by reducing lipid peroxidation, vascular permeability, and platelet aggregation.

**Figure 4:**
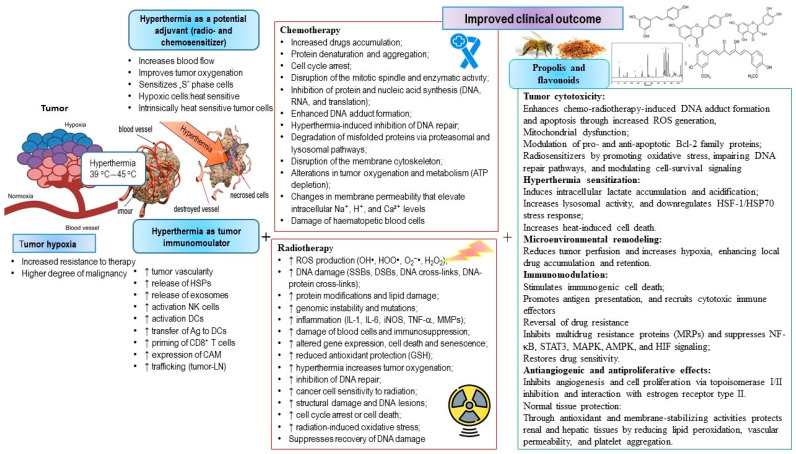
Synergistic antitumor mechanisms of hyperthermia–flavonoid therapy in combination with chemo- and radiotherapy. Combined treatment with flavonoids and chemo-radiotherapy potentiated by hyperthermia, exerts multifaceted synergistic effects that enhance tumor destruction while minimizing systemic toxicity. Combined treatment exerts potent synergistic antitumor effects through multiple, interconnected mechanisms. **(1)** *Tumor cytotoxicity*: Flavonoids enhance chemo- and radiotherapy-induced DNA damage, apoptosis, and cell cycle arrest by promoting mitochondrial dysfunction, reactive oxygen species (ROS) generation, and modulation of Bcl-2 family proteins. **(2)** *Hyperthermia sensitization*: Flavonoids induce intracellular lactate accumulation, acidification, and lysosomal activation, while suppressing HSF1/HSP70 signaling, thereby sensitizing tumor cells to heat-induced stress and apoptosis. **(3)** *Tumor microenvironment modulation*: Flavonoids improve drug penetration by remodeling the TME, reducing tumor blood flow, and enhancing hypoxia. **(4)** *Immunomodulation*: Hyperthermia and chemo-radiotherapy synergistically augment immunogenic cell death and promote recruitment of antitumor immune effectors. **(5)** *Reversal of drug resistance*: Flavonoids inhibit multidrug resistance proteins (MRPs) and key survival pathways (NF-κB, STAT3, MAPK, AMPK, and HIF), amplifying drug efficacy. **(6)** *Antiangiogenic and antiproliferative effects*: Combined treatment suppresses angiogenesis and tumor cell proliferation via topoisomerase inhibition and interference with estrogen receptor β-mediated signaling. **(7)** *Protection of normal tissues*: Through strong antioxidative and membrane-stabilizing properties, flavonoids protect renal and hepatic tissues from drug or radiation-induced oxidative injury and vascular damage. *Abbreviations*: AMPK, AMP-activated protein kinase; CAMs, cell adhesion molecules; DCs, dendritic cells; DSBs, DNA double-strand breaks; GSH, glutathione; HIF, hypoxia-inducible factors; HSF1, heat shock transcription factor 1; HSP, heat shock protein; HSPs, heat shock proteins; IL-1, interleukin-1; IL-6, interleukin-6; iNOS, inducible nitric oxide synthase; LN, lymph node; MAPK, mitogen-activated protein kinase; MMPs, matrix metalloproteinases; NF-κB, nuclear factor kappa-light-chain-enhancer of activated B-cells; NK cells, natural killer cells; ROS, reactive oxygen species; SSBs, single-strand breaks; STAT3, signal transducer and activator of transcription 3; TNF-α, tumor necrosis factor alpha.

A schematic overview of the synergistic antitumor mechanisms of natural compounds and flavonoids components in combination with HT in chemo- and radiosensitization and modulation of the immune response within the TME is presented in Figure 4.

### Challenges and Research Needs

Despite the promising therapeutic potential of the multimodal approach, several challenges must be addressed before successful clinical translation. Further research is required to determine optimal HT parameters, including temperature range, duration, treatment sequencing relative to RT, flavonoid concentrations, and the type of cells or tumor models used. Considerable variability in experimental design currently complicates comparability, synthesis, and translational relevance, while preclinical and clinical evidence remains limited. Comprehensive studies assessing safety, adverse effects, pharmacokinetics, and dose optimization are therefore urgently needed.

A major technical challenge is the lack of reliable, real-time, non-invasive temperature-monitoring technologies and the difficulty of achieving precise thermal control, particularly in deep tumors. Additionally, the influence of tumor-specific genetic and epigenetic alterations on treatment efficacy remains insufficiently explored.

Nevertheless, the observed enhancements of anti-PD-1/PD-L1 responses, PARP inhibition, suppression of HSP and HDAC activity, inhibition of angiogenesis, and robust immunomodulatory effects mediated by flavonoids provide encouraging results. Inhibition of HSP70 and HSP90 may also help overcome resistance to apoptosis. HSP70/90 inhibitors potentiate TRAIL-induced apoptosis in tumor cells. Furthermore, dietary flavonoids have been reported to induce apoptosis of cancer cells by inhibiting HSF1 activity through blocking its binding to the HSP70 promoter [13,152,153,270].

HT is a potent sensitizer of cell killing by ionizing radiation. However, it simultaneously induces HSP70 expression, which is closely associated with radioresistance. Given that HSP70 interacts with the telomerase complex. Agarwal et al. [237] demonstrated that the inhibition of telomerase activity enhances HT-mediated radiosensitization. Flavonoids therefore represent promising therapeutic options due to their ability to inactivate telomerase and suppress cancer cell proliferation. Increased tumor cell killing following combined heat and IR exposure in telomerase-inhibited cells correlates with delayed appearance and disappearance of γ-H2AX foci and impaired chromosome repair. These results suggest that telomerase inactivation prior to combined HT and RT may substantially improve tumor killing. Accordingly, deeper insight into telomerase regulation and activity might lead to novel therapeutic strategies.

Previous studies have also demonstrated the high sensitivity of leukemic cells to HT [302,303]. Deezagi et al. [304] reported that telomerase activity of hematopoietic stem cells is more sensitive to HT than in leukemic cell lines. If the inhibition of telomerase activity following HT reduces telomere length in hematopoietic progenitors, HT could represent a selective approach for treating diseases associated with uncontrolled proliferation of leukemic and cancer stem cells [305,306,307]. In that regard, the administration of flavonoids may further enhance the inhibition of telomerase activity and induce tumor cell death by apoptosis [13,39,83]. These findings suggest that combined flavonoid–HT strategies targeting telomere and telomerase dynamics may open new therapeutic avenues for leukemia treatment [13,39,83,308,309,310].

Additionally, nanoparticle-based delivery systems capable of intensifying the hyperthermic effect may substantially increase tumor destruction and therapeutic precision [48,55,111,112,147,167,180,209,218,241,291]. Future research should also focus on elucidating epigenetic alterations, defining the functional roles of immune cell subsets, and their polarization dynamics, with particular emphasis on macrophage plasticity, under HT-induced changes in the TME. Overcoming thermoresistance should be examined comprehensively, while attention should be directed toward the development of novel thermosensitizers and optimization of HT treatment protocols [139,143,169,170].

Finally, elevated temperatures not only disrupt DNA repair mechanisms, leading to replication errors, cell cycle arrest, and apoptosis, but may also modulate the composition and functional activity of the intestinal microbiota [311,312,313]. The effects of various forms of HT, including HIPEC, deep regional HT, and whole-body HT, influence the gut microbiome in ways that remain insufficiently understood. Further studies are needed to clarify beneficial versus detrimental HT-induced microbiome alterations and to explore strategies for microbiota modulation through diet, probiotics, or prebiotics. In this context, the documented beneficial effects of flavonoids on gut microbiota may help maintain intestinal homeostasis and an effective immune response.

## 7. Conclusions

Current preclinical and clinical evidence demonstrates that HT acts as a potent chemosensitizer and radiosensitizer by directly eliminating hypoxic tumor cells, enhancing cytotoxicity and apoptosis, inhibiting DNA repair mechanisms, improving tumor oxygenation, and modulating the TME. HT also promotes antitumor immunity by inducing immunogenic cell death, increasing HSP expression, enhancing antigen presentation, and improving immune cell infiltration. Moreover, HT may overcome resistance to chemotherapeutic agents by enhancing drug uptake, improving tumor perfusion, and modifying membrane permeability and cellular metabolism.

Flavonoids exhibit complementary anticancer activities through the induction of cell cycle arrest, suppression of proliferation, induction of apoptosis, inhibition of metastasis and angiogenesis, modulation of oncogenic signaling pathways and epigenetic regulation. In addition to their direct effects on tumor cells, polyphenols influence macrophage polarization, glucose metabolism, and gut microbiota composition. When combined with HT or standard oncologic therapies, especially in nanoformulated forms, flavonoids demonstrate synergistic antitumor effects. Emerging evidence indicates that HT–flavonoid combinations may enhance responses to immune checkpoint inhibitors (anti-PD-1/PD-L1), PARP inhibitors, and HDAC inhibitors. Furthermore, the suppression of HSP70 and HSP90 may reverse apoptosis resistance and potentiate TRAIL-induced tumor cell death. Nanoparticle-based systems capable of controlled heating and targeted flavonoid delivery therefore hold significant potential to amplify therapeutic efficacy and improve clinical outcomes.

Despite the limited number of studies and the considerable knowledge gap concerning the combined application of natural compounds with chemo-RT and HT, available evidence suggests that each modality may enhance the efficacy of the others. This synergism likely occurs through the induction of DAMPs, including HSPs, calreticulin, and HMGB1; modulation of the immune microenvironment; suppression of HSP-mediated thermotolerance; and amplification of ROS-driven cellular stress. Collectively, these processes promote apoptosis and immunogenic cell death, thereby indirectly enhancing antigen presentation. Future research should focus on development and rigorous evaluation of integrative therapeutic strategies, with particular attention to optimal dosing, treatment timing, and potential interactions.

## Figures and Tables

**Table 1:** Physiological and molecular effects of hyperthermia.

Effect on Cellular Components	Mechanism Action and Functional Alterations	References
Cell membrane	− changes in fluidity/stability	[3,4,5,8,9,10,11,12]
	− structural changes	
	− alterations in ion transport (Ca^2+^, Na^+^, Mg^+^, K^+^)	
	− changes in cytosolic proteins and channels	
Cytoplasm	− changes and denaturation in protein structure and function	
	− protein synthesis disorder	
	− protein aggregations	
	− induction of HSP synthesis (HSP27, HSP40, HSP60, HSP70, HSP90)	
Mitochondria	− ↑ permeability of inner mitochondrial membrane	
	− depolarization of mitochondrial membrane	
	− ATP reduction	
Endoplasmic reticulum (ER)	− ↑ in ER stress due to the intensive accumulation of misfolded proteins	
Nucleus	− impairment of DNA/RNA synthesis, − ↓ activity of DNA repair enzymes − changes in gene expression and signaling − conformational DNA change	
Effects of hyperthermia on the body level	− ↑ metabolic rate by 8–10%	[3,4,5,6,7,8,14,15,16,17,18,19,20,21,22,23,24,25]
	− ↑ minute volume	
	− ↑ PaO_2_ (by 5 mmHg) and PaCO_2_ (by 2 mmHg) per each degree above 37 °C	
	− ↓ affinity of hemoglobin for oxygen	
	− increased cardiac output	
	− ↓ central venous pressure and systemic vascular resistance (↑ blood flow to the skin and ↓ central blood volume through vasodilation)	
	− ↑ redistribution of blood flow to skin (up to 50–70% of the cardiac output)	
	− reduced glomerular filtration and impaired renal drug clearance	
	− ↓ hepatic blood flow and hepatic drug clearance by 50–75%	
	− ↑ permeability due to capillary endothelial dysfunction	
	− cognitive impairment	
	− hemoconcentration and hypokalemia due to electrolyte loss	
	− impaired intestinal motility	
	− ↑ permeability of gastrointestinal mucosa	
	− blood hypercoagulability	
	− **Improved immune cell function** − *Innate immunity*: − ↑ recruitment of neutrophils from the bone marrow − ↑ neutrophil migration to local sites of infection − ↑ activity of NK cells − ↑ bacterial clearance by resident tissue macrophages − ↑ migration of dendritic cells to lymphoid tissues − *Adaptive immunity*: − ↑ activity of antigen-presenting dendritic cells − ↑ circulation of lymphocytes through lymphoid tissue − promoted T-cell proliferation − ↓ threshold for T-cell signaling	[2,4,6,13,19,25]
	− impaired microbial growth for some pathogens	

*Abbreviations*: ATP, adenosine triphosphate; Ca^2+^, calcium ion; DNA, deoxyribonucleic acid; ER, endoplasmic reticulum; HSP, heat shock protein; K^+^, potassium ion; Mg^2+^, magnesium ion; Na^+^, sodium ion; RNA, ribonucleic acid. ↑ increase; ↓ decrease.

**Table 2:** Hyperthermia as a potential adjuvant.

Hyperthermia as a Potential Adjuvant	Molecular Mechanisms	References
Cell-killing effects:	− cell cycle arrest − cell death − ↑ in quiescent cell repopulation	[1,4,5,8,17]
Vascular effects:	− ↑ vasodilatation − ↑ perfusion − ↑ permeability − ↑ reoxygenation − ↓ hypoxia	[8,9,10,11,12,13,14,15,18]
Genomic effects:	− ↑ **DNA breakage—direct and indirect effects**: − *Direct effects*: DDR activation and decelerated DNA replication and repair − DNA breaks (SSBs, DSBs) − γH2AX foci formation − ATM autophosphorylation − ↓ DNA polymerase activity − ↓ DNA topoisomerase activity − *Indirect effect*: DDR activation and/or ARF induction − **↑** ROS production ⟶ OS ⟶ DNA damage − cell cycle arrest − cell cycle checkpoint activation: the G1, G2/M and the metaphase checkpoint ⟶ ATM and ATR activation − decelerated DNA replication ⟶ **↑** DNA damage − **↑** E2F1 ⟶ cell death induction − **↓ repair inhibition (direct and indirect effects)** − base excision repair (BER) − mismatch repair (MMR) − nucleotide excision repair (NER) − translesion DNA synthesis (TLS) − non-homologous end joining (NHEJ) − homologous recombination (HR)	[8,9,10,17,18,19,20,25]
Immune effects	− **↑ immune stimulation**: − **NK cells:** ↑ NKG2D receptor, ↑CD94 and CD56, ↑ proliferation, ↑ activity on tumor cells − **dendritic cells**: ↑ proliferation, ↑ maturation via heat shock transcription factor 1 (HSF1), HSP70, and Toll-like receptor (TLR), ↑ activation of primary T- or B-cells, ↑ TLRs, ↑CD11c, ↑antigen presentation, ↑Th cells maturation, ↓ oxidative phosphorylation,↑ glycolysis and ROS production, ↑ DC metabolic reprogramming − **macrophages:** ↑oxidative stress, ↑ iNOS and NO, ↑ activation of NF-κB pathway, ↑ CXCLs and ILs, ↑ phagocytosis, ↑ M1-type cells—strong antitumor responses − **T- and B-cells**: ↑ T-cell migration, ↑cytotoxic T-cell population, ↑ Fas ligand, ↑ granzyme B and perforin, NF-κB activation, ↑ B-cell proliferation and activation, B-cells activate DCs or provide antigens for the activation and replication of CD4+ and CD8+ T-cells − **cytokine production:** ↑ cytokines such as IL-1β, IL-6, IL-8, IL-10, IFNγ, TNFα and CCL22 − adhesion molecules: ↑ L-selectin, P-selectin, and intercellular adhesion molecule-1 in the vessel wall − **MHC molecules**: ↑ expression of MHC class II molecules (e.g., HLA-DR) on the surface of T- and B-cells − heat shock proteins (HSPs): ↑ HSP70 and HSP90	[2,4,6,13,23,25]
Effects on TME	− **Hypoxia**: − hyperthermia reduces hypoxia by enhancing tumor oxygenation through increased blood flow − extended hyperthermia may intensify hypoxia by inducing vascular closure − **pH**: hyperthermia can counteract low pH by increasing blood flow and improving oxygenation, increasing efficacy of combined therapies − **GSH**: ↓ GSH production, ↑ increased GSH depletion; some tumors may boost GSH production and decrease effectiveness of cancer treatment − **Angiogenesis**: increased hyperthermia (>41 °C) damages blood vessels and promotes their closure − **ROS:** ↑ metabolic activities and mitochondrial dysfunction, ↑ ROS production, ↑ oxidative stress and cell damage, ↑ cell death	[13,20,23,25]
	− improvement in macromolecular and liposomal drug delivery	
Effects on tumor metabolism and oxygenation	− ↑ in lactate concentration	[1,3,4,5,8,13,14,15,16,17,18,19,20,24,25,26]
	− improved tumor oxygenation (median temperatures < 44 °C)	
	− ↓ tumor oxygenation (>44 °C for 60 min led to decreased oxygenation)	
	− ↑ temperatures increase ROS production within mitochondria	
	− mitochondrial thermal damage and excessive oxidative stress	
	− disrupting ATP production and limiting the cell’s energy supply	
	− necrosis and ATP depletion result in inflammation	
	− ↑ protein misfolding and aggregation, triggering cellular stress responses such as unfolded protein response and HSP expression	
	− prolonged hyperthermia induces autophagy-related cell death and inflammation	
	− ↑ urinary markers such as neutrophil gelatinase-associated lipocalin (NGAL), kidney injury molecule-1 (KIM-1), and liver fatty acid-binding protein (L-FABP)	
	− endothelial dysfunction	
	− short-term neurological dysfunction and longer-lasting cognitive deficits	
	− inflammation and damage to neurons	

*Abbreviations*: ARF, alternative reading frame tumor suppressor; ATM, ataxia–telangiectasia mutated kinase; ATR, ataxia–telangiectasia and Rad3-related kinase; CCL22, C–C motif chemokine ligand 22; CXCLs, CXC motif chemokines; DDR, DNA damage response; DSBs, DNA double-strand breaks; E2F1, E2 promoter binding factor 1; γH2AX, phosphorylated histone H2AX; GSH, glutathione; HSF1, heat shock transcription factor 1; HSP, heat shock protein; iNOS, inducible nitric oxide synthase; NKG2D, natural killer group 2D receptor; ROS, reactive oxygen species; SSBs, single-strand breaks; TLR, Toll-like receptor; TME, tumor microenvironment. ↑ increase; ↓ decrease.

**Table 3:** Types of hyperthermia, clinical applications and types of energy delivered to tumors.

Types of Hyperthermia			
Local HT:	Regional HT:	Whole-Body HT (Systemic)	References
− superficial − intracavital − intraluminal − intersticial	− abdominal − pelvic − limbs		[46,47,48,49,50,51,52,53]
**Type/site of tumor**			
− superficial tumor − intracavital tumor − intraluminal tumor − intersticial tumor	− deep-seated tumor − locally advanced tumor	− disseminated/metastatic diseases	[46,47,48,49,50,51,52,53]
**Clinical application**			
− head and neck cancer − breast cancer − rectal cancer − esophageal cancer − soft tissue sarcomas − malignant glioma	− rectal cancer − cervical cancer − bladder cancer − prostate cancer − soft tissue sarcomas − ovarian cancer − peritoneal carcinomatosis − mesothelioma	− malignant melanoma − recurrent soft tissue sarcomas − ovarian cancer	[46,47,48,49,50,51,52,53]
**Type of energy/equipment**			
− microwaves (MV) − radiofrequency (RF) − ultrasound (US)	− microwaves (MV) − radiofrequency (RF) − ultrasound (US)	− infrared radiators − hot water blankets − thermal chamber	
**Hot sources**			
− hot water perfusion − resistive wire implants − ferromagnetic implants − nanoparticles	− hot water perfusion − resistive wire implants − ferromagnetic implants − nanoparticles		[46,47,48,49,50,51,52,53]

**Table 4:** Temperature-dependent effects of hyperthermia on angiogenesis in cancer.

Mild Hyperthermia	Moderate Hyperthermia	Thermal Ablation
↑ blood perfusion into tumors	↑ heat shock and inhibition of DNA repair mechanisms	↑ necrotic cell death
↓ DNA repair mechanisms	↑ replication errors	↑ release of tumor antigens
↑ sensitization for CT and RT	↑ cell cycle arrest	↑ antigen presentation
↑ blood flow	↑ apoptosis	↑ antitumor immune response
↑ oxygenation	↓ blood flow	↓ blood flow
↑ efficacy of chemotherapy and RT	↑ hypoxia	↑ hypoxia
↑ antitumor immune response	↑ metabolism	↓ DNA repair
↑ death by mitotic catastrophe	↓ DNA repair	↑ DNA damage
	↑ DNA damage	
	↑ antitumor immune response	

↑ increase; ↓ decrease.

**Table 5:** Advantages and disadvantages of hyperthermia in combination with chemotherapy and RT.

Modality	Advantages	Disadvantages	References
Thermoradiotheraphy	− Strong synergistic effect − Reduced radiation doses − Enhanced radiosensitization through increased blood perfusion and inhibition of DNA repair mechanisms − Cell cycle blockage − Inhibition of tumor VEGF production − Stimulation of antitumor immune response − Minor acute side effects	− Increased damage to normal tissues − Pain control − Antagonistic effect on drug delivery due to vessel stasis, hypoxia and low pH	[85,97,99,102,103,104]
Thermochemotheraphy	− Increased chemosensitivity − Reduction in drug resistance − Increased drug uptake − Increased reversible changes in endothelial cells (ECs), disrupting cell–cell junctions (VE-cadherin) and F-actin, significantly increasing permeability (selectively increased the size of fenestrations—hyperpermeability) − Reduce dose of chemotherapeutics − Reduced body damage − Increased cellular death and necrosis without increasing its oncogenic potential (hyperthermia kill cancer cells, does not promote mutations that lead to cancer) − Reduction in interstitial fluid pressure—improved drug delivery − Increased drug cytotoxicity − Cost savings of chemotherapy treatment − Metastasis prevention and control − Increased antitumor immune response	− Increased drug toxicity to normal tissue − Thermotolerance − Antagonistic effect on drug delivery due to vessel stasis, hypoxia and low pH	
Themochemo-radiotheraphy	− Synergistic effects—increased cell sensitivity to therapy by impairing DNA repair, increasing drug uptake, and alteration to the tumor microenvironment (like improving oxygenation) − Down-staging − Improved resection rate − Reduced advanced-stage cancer − Increased survival and disease-free state	− Toxic reaction − Long treatment course − Hematological toxicity	

## Data Availability

No new data were created or analyzed in this study. Data sharing is not applicable to this article.

## Abbreviations

The following abbreviations are used in this manuscript:

AIF	Apoptosis-inducing factor
AP-1	Activator protein-1
APCs	Antigen presenting cells
bFGF	Basic fibroblast growth factor
CAAs	Cancer-associated fibroblasts
CAFs	Cancer-associated fibroblasts
CP	Cisplatin
CPL	*Caucalis platycarpos*
CSC	Cancer stem cell
CTLs	Cytotoxic T-lymphocytes
DCs	Dendritic cells
EAT	Ehrlich ascites tumor
EGCG	Epigallocatechin gallate
EGFR	Epidermal growth factor receptor
EMT	Epithelial–mesenchymal transition
EPR	Enhanced permeability and retention
ER	Endoplasmic reticulum
ERK1/2	Extracellular signal-regulated kinase 1/2
GM-SCF	Granulocyte-macrophage colony-stimulating factor
GSH	Glutathione
H_2_0_2_	Hydrogen peroxide
HDAC	Histone deacetylase
HIF-1	Hypoxia-inducible factor-1
HIPEC	Hyperthermic intraperitoneal chemotherapy
HMT	Hematoxylin
HR	Homologous recombination
HSEs	Heat shock elements
HSF1	Heat shock factor 1
HSPs	Heat shock proteins
HT	Hyperthermia
IFN	Interferon
IFP	Interstitial fluid pressure
IL	Interleukin
ILS	Increased lifespan
iNOS	Inducible nitric oxide synthase
MAPK	Mitogen-activated protein kinase
MDR	Multidrug resistance
MDSCs	Myeloid-derived suppressor cells
mEHT	Modulated electro-hyperthermia
MHT	Magnetic hyperthermia
MIH	Magnetic induction hyperthermia
MMP	Mitochondrial membrane potential
MMPs	Matrix metalloproteinases
MMP2	Matrix metalloproteinase 2
MMP9	Matrix metalloproteinase 9
MNs	Micronuclei
mTOR	Mammalian target of rapamycin
NAR	Naringenin
NC	Nanocrystalline
NETs	Neutrophil extracellular traps
NF-κB	Nuclear factor kappa B
NHEJ	Non-homologous end joining
NK	Natural killer
NO	Nitric oxide
NPs	Nanoparticles
PAMPs	Pathogen-associated molecular patterns
PARP	Poly (ADP-ribose) polymerase 1
PI3K/Akt	Phosphatidyl inositol-3-kinase (PI3K)/Akt
PTAs	Photothermal agents
PTT	Photothermal therapy
QU	Quercetin
ROS	Reactive oxygen species
RT	Radiotherapy
SOD	Superoxide dismutase
TAMs	Tumor-associated macrophages
TAN	Tumor-associated neutrophil
TASCs	Tumor-associated stromal cells
TANCs	Tumor-associated natural killer cells
TC-HT	Thermal cycling-hyperthermia
TGF	Tumor growth factor
TLRs	Toll-like receptors
TNF-α	Tumor necrosis factor alpha
TME	Tumor microenvironment
VEGF	Vascular endothelial growth factor
WSDP	Aqueous propolis solution
